# NIR-responsive electrospun nanofiber dressing promotes diabetic-infected wound healing with programmed combined temperature-coordinated photothermal therapy

**DOI:** 10.1186/s12951-024-02621-2

**Published:** 2024-07-01

**Authors:** Jinlang Fu, Ding Wang, Zinan Tang, Yixin Xu, Jiajun Xie, Rong Chen, Pinkai Wang, Qiang Zhong, Yanhong Ning, Mingyuan Lei, Huaming Mai, Hao Li, Haibing Liu, Jian Wang, Hao Cheng

**Affiliations:** 1 Department of Orthopedic, Nanfang Hospital, Southern Medical University, Guangzhou, 510515 China; 2 Department of Orthopaedic, Affiliated Hengyang Hospital of Hunan Normal University & Hengyang Central Hospital, Hengyang, Hunan 421001 China; 3 Department of Orthopedics, The Second Affiliated Hospital of Nanchang University, Nanchang University, Nanchang, 330006 China

**Keywords:** Mxene, Nanofiber membrane, Programmatic photothermal property, Antibacterial activity, Wound healing

## Abstract

**Background:**

Diabetic wounds present significant challenges, specifically in terms of bacterial infection and delayed healing. Therefore, it is crucial to address local bacterial issues and promote accelerated wound healing. In this investigation, we utilized electrospinning to fabricate microgel/nanofiber membranes encapsulating MXene-encapsulated microgels and chitosan/gelatin polymers.

**Results:**

The film dressing facilitates programmed photothermal therapy (PPT) and mild photothermal therapy (MPTT) under near-infrared (NIR), showcasing swift and extensive antibacterial and biofilm-disrupting capabilities. The PPT effect achieves prompt sterilization within 5 min at 52 °C and disperses mature biofilm within 10 min. Concurrently, by adjusting the NIR power to induce local mild heating (42 °C), the dressing stimulates fibroblast proliferation and migration, significantly enhancing vascularization. Moreover, in vivo experimentation successfully validates the film dressing, underscoring its immense potential in addressing the intricacies of diabetic wounds.

**Conclusions:**

The MXene microgel-loaded nanofiber dressing employs temperature-coordinated photothermal therapy, effectively amalgamating the advantageous features of high-temperature sterilization and low-temperature promotion of wound healing. It exhibits rapid, broad-spectrum antibacterial and biofilm-disrupting capabilities, exceptional biocompatibility, and noteworthy effects on promoting cell proliferation and vascularization. These results affirm the efficacy of our nanofiber dressing, highlighting its significant potential in addressing the challenge of diabetic wounds struggling to heal due to infection.

**Graphical Abstract:**

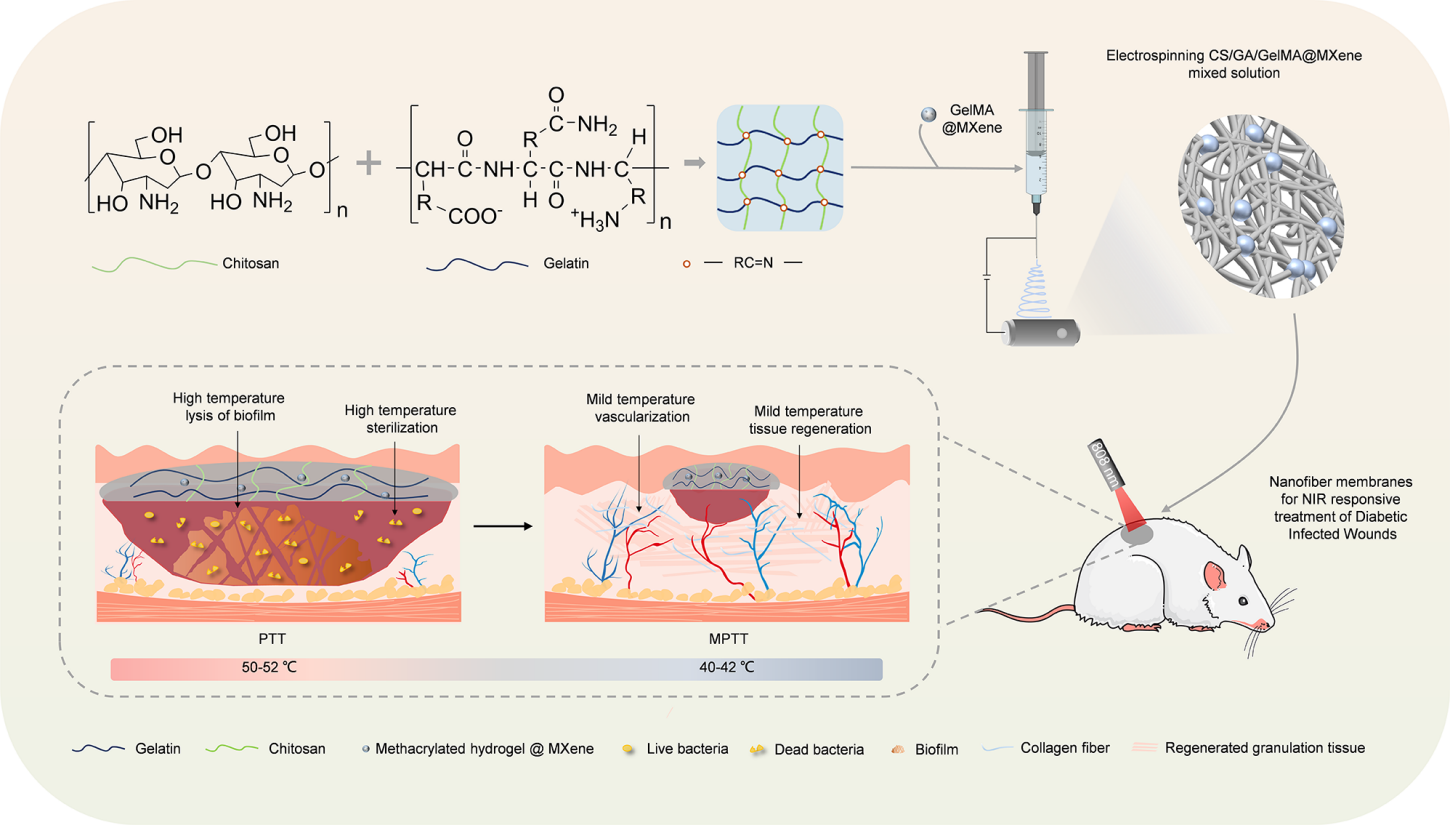

**Supplementary Information:**

The online version contains supplementary material available at 10.1186/s12951-024-02621-2.

## Background

Diabetes, a complex metabolic disorder, profoundly impacts the well-being of a significant global populace [1]. Currently, around 285 million adults aged 20 to 79 worldwide are living with diabetes, and the number is projected to reach 439 million by 2030 [2]. Individuals with diabetes are at an elevated risk of developing diabetic wounds, representing a substantial public health issue (4-10%) [3]. The healing of diabetic wounds is often complicated and extended by infection and ischemia [4]. Infection and subsequent biofilm development usually result in extensive destruction of skin tissue structure and prolonged wound healing [5]. Insufficient blood supply due to vascular injuries in hyperglycemia, results in cells and tissues experiencing a deficiency of oxygen and nutrients [6]. Several biofunctional wound dressings have been developed to concurrently address both infection control and improved angiogenesis [7]. However, these dressings predominantly depend on bactericidal agents and growth factors to achieve their respective functions, which can lead to a heightened risk of antibiotic resistance during biofilm treatment and necessitate a complex controlled-release design system to sustain the bioactivity of growth factors at therapeutic levels [8]. As a result, there is a pressing need to engineer a novel wound dressing that can effectively eradicate local bacteria, disperse mature biofilms, and simultaneously promote vascularization using alternative methodologies.

Diverse wound dressing products (such as hydrogels, sponges, and films) have been developed for infected wound treatment [9]. Well-organized nanofiber scaffolds fabricated by electrospinning have garnered attention due to their ability to emulate the fibrous structure of the extracellular matrix (ECM), thereby promoting cell proliferation and migration [10]. These nanofiber membranes are characterized by an exceptionally large specific surface area and superior permeability to gases and liquids [11]. The versatility of electrospinning is noteworthy given the adaptability of material choices and convenience of preparation methodologies. Chitosan (CS), is a linear polysaccharide known for recognition as a promising wound dressing material due to its natural origin, abundant availability, cost-effectiveness, absorbency, and non-immunogenic properties [12]. However, the low solubility, inherent hydrophobicity, and inadequate mechanical strength of CS present challenges in maintaining stable fiber structures during electrospinning [13]. The protracted degradation rate of CS may cause a discordance with the body’s skin regeneration pace, potentially leading to healing delays [13]. To circumvent these limitations, gelatin (GA), a naturally occurring protein polymer with high biocompatibility, was incorporated into CS during the electrospinning process to create a composite GACS nanofiber membrane. Owing to its superior water solubility and electrospinnability, GA expedites the electrospinning process [14]. Furthermore, the abundance of hydrogen bond donors and acceptors in GA molecules augments the hydrophilicity of the electrospun dressing surface, thereby promoting cell adhesion and proliferation [15]. Therefore, the inclusion of GA into CS matrices as a supplementary polymer could equip the wound dressing with the desired physicochemical properties.

Photothermal therapy (PTT) delivers localized thermal treatment via non-invasive light exposure, possessing the ability to compromise the structural integrity of pathogenic bacteria [16]. Significantly, bacteria find it challenging to develop resistance to PTT, in contrast to their reaction to antibiotics, due to their inability to suppress or decrease the absorption of heat [17]. Furthermore, thermal therapy could dismantle the biofilm structure by deactivating the inherent bioactive matrix, composed of nucleic acids and proteins, thus eradicating bacteria that are otherwise protected [18]. At present, a variety of photothermal materials have been employed for the extermination of bacteria, including carbon-based constituents, noble metal nanoparticles, conjugated polymers, and assorted organic frameworks [19]. Among these, a unique two-dimensional nanomaterial, MXene, is noteworthy due to its exceptional photothermal conversion efficiency [20]. MXene primarily consists of elements such as carbon, nitrogen, and titanium, it possesses good biocompatibility and can undergo degradation and subsequent elimination from the body [21]. However, limitations such as subpar stability and vulnerability to oxidation have restricted its use in the biomedical arena [22]. Herein, to augment the stability of MXene at the site of the wound, we suggest employing physical encapsulation by encasing MXene within GA Methacryloyl (GelMA) microspheres. The encapsulation of GelMA microspheres not only furnishes a protective layer, reducing oxidation in the environment but also enhances the mechanical stability of the composite material [23]. Thus, this encapsulation strategy is expected to extend the enduring and realized efficient application of MXene in wound dressing for fighting against bacteria contamination [24].

Simultaneously, the challenge of inadequate blood perfusion, stemming from vascular system damage due to prolonged elevated glucose levels, remains a significant obstacle in the treatment of difficult-to-heal wounds [25]. Bioactive molecules such as vascular endothelial growth factor (VEGF) and matrix metalloproteinases (MMPs) have been utilized to initiate angiogenesis in this context [26]. However, maintaining their bioactivity for extended treatment periods is challenging, as these substances are generally vulnerable to environmental factors, including temperature and pH [27]. As a result, these agents are frequently encapsulated into drug delivery systems, but achieving a consistent sustained release and maintaining a therapeutic concentration still presents a challenge [28]. Recently, mild photothermal therapy (MPTT, 40–42 °C) has emerged as a potential strategy to promote tissue regeneration and growth, providing precision, rapidity, durability, and maintainability that surpasses the use of bioactive agents [29]. Mild heat treatment has been demonstrated to elevate the expression of genes and Heat shock protein related to angiogenesis, thereby stimulating the formation of endothelial cell tubes, enhancing vascularization, and increasing the density of newly formed vessels in granulation tissue [30]. Thus, the application of MXene-GelMA photothermal microspheres not only achieves PTT sterilization but also incorporates MPTT angiogenesis. Through simple adjustments of near-infrared (NIR) light parameters, this temperature-regulated PTT can be implemented in a programmable fashion, thereby showcasing its efficacy in enhancing the healing of infected diabetic wounds [31]. The programmed use of PTT in wound dressing has not been reported yet.

In this study, we engineered a NIR-responsive electrospun nanofiber dressing, incorporating Mxene encapsulated GelMA microgels. The GA modified CS nanofiber membrane was projected to exhibit enhanced physicochemical properties, such as facile electrospinning, increased hydrophilicity, degradability, and robust mechanical resilience [32]. Under NIR irradiation, the nanofiber membrane can execute programmed photothermal operations. Broad-spectrum sterilization and biofilm lysis can be accomplished through high-temperature photothermal treatment, whereas low-temperature photothermal treatment can enhance blood perfusion and stimulate vascular formation. These combined effects would contribute to the accelerated healing of diabetic wounds and highlight the translation potential in the management of infected diabetic wounds.

## Materials and methods

### Materials

Gelatin (99% biotech grade), chitosan (CS, viscosity 100–200 mpa.s, ≥ 95% deacetylated), phalloidin-TRITC(C_60_H_70_N_12_O_13_S_2,_ MW 1231.4), iFluor 488 Phalloidin, CCK-8 Cell Viability Reagent, 4’,6-diamidino-2-phenylindole (DAPI) and phosphate-buffered saline (PBS) were purchased from Macklin (Shanghai, China). Ti_3_AlC_2_ ceramic powders were purchased from Beijing Forsman Technology (China). Hydrogen fluoride (HF, 40%), and dimethylsulfoxide (DMSO) were purchased from J&K Chemical (Shanghai, China) without further treatment. Reagents of unspecified purity were analytically pure. The high-voltage power supply of the electrospinning machine, single-channel syringe pump, X-Y axis motion system, and drum receiving device were all purchased from Foshan Lepton Precision Measurement and Control Technology Co., Ltd., Guangdong, China. The release paper was purchased from Spinning Intelligent Equipment Co., Ltd., Qingdao, China. Fourier transform infrared spectroscopy(FT-IR) instrumentation (Thermo Scientific Nicolet iS10 infrared FT-IR spectrometer, Massachusetts, USA). L929 rat cells were sourced from Cyagen (Jiangsu, China). *E. coli* (ATCC 252,922), MRSA (ATCC 43,300), and *S. aureus* (ATCC 25,923) were acquired from ATCC (Manassas, VA, USA).

### Methods

#### Preparation of Mxenes

MXene was fabricated via a chemical delamination technique, adhering to a protocol delineated in earlier research [33]. Precisely, 1 g of Ti_3_AlC_2_ powder was immersed in 10 mL of 40 wt% HF at room temperature and stirred for 18 h. Post-centrifugation, the sample was cleansed with ethanol and water before being dispersed in a 10 mL solution of dimethyl sulfoxide (DMSO) and stirred at room temperature for a span of 5 h. The sample was subsequently isolated, washed three times with water to remove any residual DMSO, and then transferred to deionized water under nitrogen conditions for a 5-hour ultrasonication procedure. Following another round of centrifugation and washing with ethanol and water, the unadulterated Ti_3_C_2_T_x_ nanosheets were procured. To optimize the nanosheets’ interaction area with NIR light and escalate photothermal conversion efficiency, the Ti_3_C_2_T_x_ nanosheets underwent an additional 12-hour sonication process utilizing an ultrasonic disruptor, resulting in the formation of a dispersion of nano fragments.

#### Synthesis of Mxene-loaded microspheres

In line with previously established methods, GelMA was synthesized [34]. In this study, gelatin was first dissolved in a phosphate buffer with a pH of 7.5, heated to 50 °C. Subsequently, methacrylic anhydride was incorporated into the solution under intense stirring. Following a reaction period of one hour, the mixture was diluted and subjected to dialysis against distilled water at 40 °C for a duration of 24 h. Finally, the reaction product was freeze-dried, yielding gelatin methacrylamide as a white solid. The fabrication of GelMA microgels was executed through a microfluidic emulsion technique using a coaxial needle and a pair of syringe pumps (TYD 01–01, Leadfluid®, China). In a brief description, the aqueous phase was formulated by integrating Mxene with a 10% w/v GelMA polymer solution, which contained a 0.5% w/v photoinitiator, Irgacure 2959. The oil phase was concocted using paraffin oil, supplemented with a 5% w/v surfactant Span 80. The aqueous and oil phases were administered with a strictly regulated water-to-oil flow rate ratio, spanning from 0.1 to 0.13 (oil phase at 60 µl/min and water phase at 6–8 µl/min). Emulsion droplets were generated and subsequently cross-linked through UV irradiation (64 mW/cm^2^, 360–480 nm) for a duration of 1 min. Lastly, the resultant cross-linked microspheres, which housed photothermal nanoparticles, were collected via centrifugation at 4000 rpm for 5 min.

#### Prepolymer solution preparation

A CS solution was concocted by dissolving 150 mg of CS (30%) in 10 mL of an 80% acetic acid solution. This solution was subjected to sonication with a high-frequency ultrasonic apparatus for 2 min and gently mixed at ambient temperature for a duration of 10 h to ensure homogeneity. Subsequently, a gelatin-chitosan (GACS) blend was formulated by dissolving 150 mg of CS (30%) and 350 mg of gelatin (GA, 70%) in 10 mL of an identical 80% acetic acid solution, which was also gently stirred at room temperature post-sonication. Before initiating the electrospinning process, MXene-infused microgels (mGelMx) at various concentrations (20 µg/ml, 40 µg/ml, 50 µg/ml, and 60 µg/ml) were amalgamated into the prepolymer solution (MGACS). Concurrently, to ascertain the optimal CS to GA ratio, different weight ratios (GA: CS, wt/wt = 2:8, 3:7, 4:6, 5:5, 6:4, 7:3) were prepared and stirred as part of the solution optimization process.

#### Electrospinning of the nanofiber membrane

**Fig. 1:**
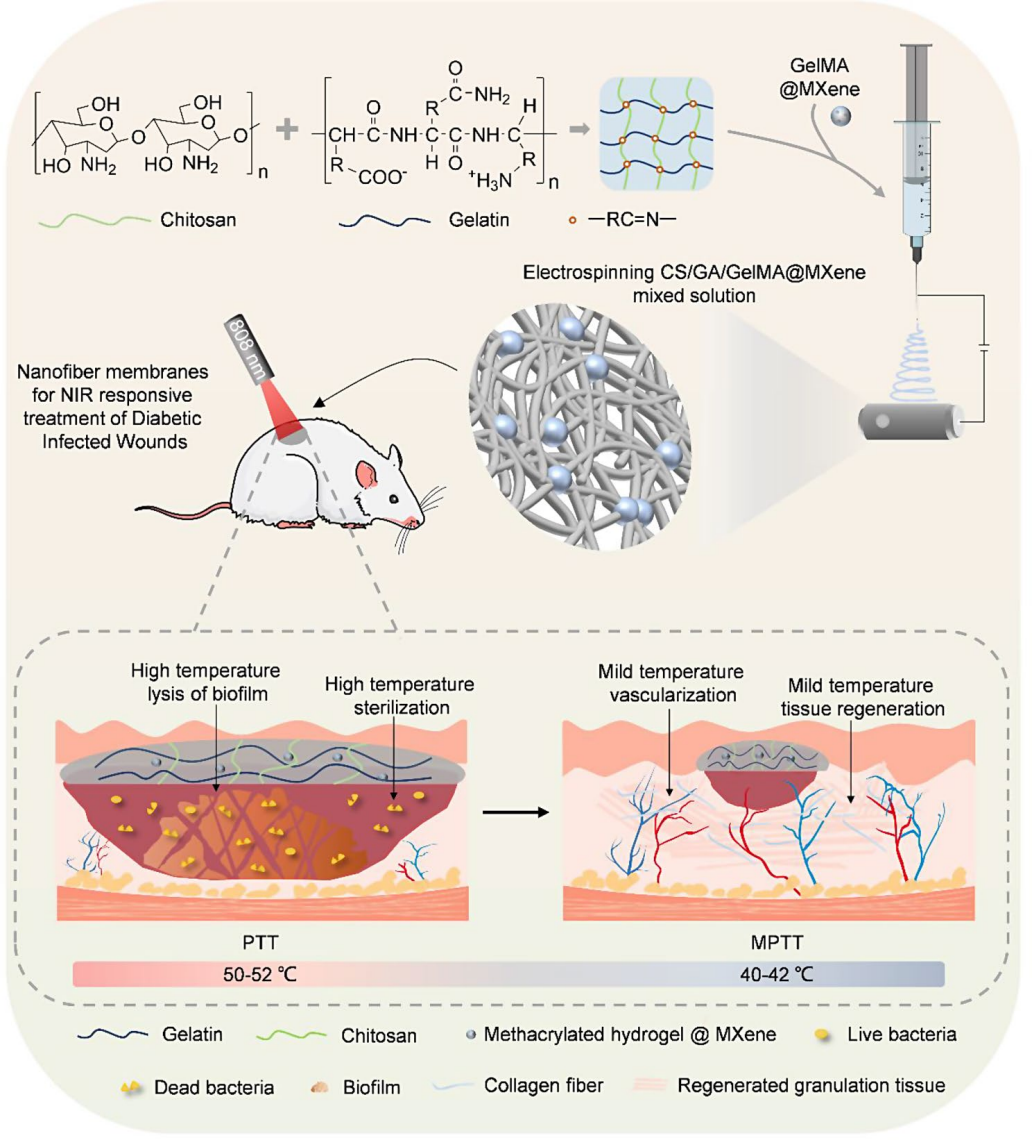
A schematic illustration of fabricating membrane dressings through the electrospinning method. This utilizes chitosan/gelatin polymer-loaded MXene-encapsulated microgels, designed to expedite the healing process of infectious diabetic wounds

Transfer the relevant prepolymer solutions (CS, GACS, and MGACS) into a 10 mL syringe connected to an 18-gauge stainless steel needle. The solutions were then extruded via the needle’s tip at a rate of 0.5 mL/h, facilitated by a syringe pump. A voltage of 20 kV was applied, with a maintained distance of 14 cm between the needle and the collection electrode. The ensuing electrospun nanofibers were deposited onto a rotating cylinder, wrapped with release paper, and rotated at a speed of 500 rpm. For a visual representation of the nanofiber fabrication technique, refer to Fig. 1.

#### Scanning electron microscopy scanning of Photothermal microspheres and electrospun nanofibers

The structure and diameter of photothermal microspheres, and microgel composite fiber membranes were scrutinized using a scanning electron microscope (ZEISS Sigma 300 SEM, Jena, Germany). Additionally, both SEM and the transmission electron microscope (Thermo Scientific Talos F200S G2S/TEM, Waltham, MA, USA) were employed to assess the morphology and size of the Mxene. Initially, MXene was dispersed in a minimal volume of absolute ethanol via sonication, followed by deposition on tin foil. The samples were subsequently dried in an oven, and the resulting dry foils were sectioned into 5 mm^2^ fragments. Regarding the photothermal microspheres, they were subjected to freeze-drying in a lyophilizer overnight before SEM and TEM analysis. To generate the microgel composite fiber membrane, the nanofiber membrane was partitioned into 3 mm squares and then subjected to a drying process. Additionally, the cross-sectional analysis of the dried composite fiber membrane was performed to assess the thickness of the nanofiber membrane. The mean diameters of Mxene, photothermal microspheres, and microgel composite fiber membranes were calculated using ImageJ software (version 1.53).

#### FT-IR analysis of the electrospun nanofibers

The Thermo Scientific Nicolet iS10 Infrared Spectrometer (MA, USA) was utilized to assess the chemical functional groups and composition of the nanofiber membranes. Initially, the electrospun samples were ground in conjunction with potassium bromide. Following this, the resultant mixed powder was compacted into a tablet using a hydraulic machine (Anatomic Instruments NLFY-60, Tianjin, China) before undergoing FT-IR analysis.

#### Swelling ratio and degradation rate assays of the electrospun nanofibers

The membrane samples were first dehydrated in a vacuum oven for a set duration of 24 h. Following this, their weights were measured using a micro-scale, and this confirmed dry weight was labeled as M_0_. These samples were then submerged in PBS)for a period of three days to ensure they achieved a state of equilibrium swelling before reweighing. The weight post-immersion was recorded as M_1_. The swelling ratio for the samples was subsequently computed with the following equation: swelling ratio = (M_1_ – M_0_) / M_0_.

The in vitro degradation rate of the electrospun nanofibers was evaluated by immersing the samples in PBS at room temperature for a duration of 14 days. These samples were periodically removed from the PBS solution at specific intervals (0, 1, 3, 5, 7, and 14 days), and all excess moisture was thoroughly eliminated. The weight recorded at each time point(W_2_), was contrasted with the initial weight (W_1_). Following this, the degradation rate was calculated using the formula: Degradation rate = [(W_1_ – W_2_) / W_1_] × 100%.

#### Contact angle analysis

The hydrophilic properties of the nanofiber membranes were investigated utilizing an automated tilt contact angle measurement instrument, the DSA-X ROLL (BETOP, Guangzhou, China). A volume of 20 µl of deionized water was carefully applied onto the surface of the fiber membrane, and high-resolution images were subsequently captured for thorough examination. Post-image acquisition, which displayed the static deposition of the liquid, an intricate analysis was conducted. Each specimen underwent a minimum of three measurements to determine the average contact angle.

#### Photothermal properties of the nanofiber membrane

Nanofibers, incorporating varying mass percentages of mGelMx (20 µg/ml, 40 µg/ml, 50 µg/ml, and 60 µg/ml), were sectioned into fragments measuring 0.6 × 0.6 cm and arranged in a 24-well plate. Subsequently, each well was supplemented with 0.5 mL of deionized water. After exposure to NIR laser irradiation (808 nm, 1 W·cm^2^), both visual representations and thermal data were recorded using a thermal imaging temperature detector. The objective of the experimental design is to scrutinize the photothermal properties of the microgel composite fiber membrane and elucidate the correlation between temperature escalation and mGelMx concentration.

Furthermore, the MGACS nanofiber mat was exposed to NIR irradiation at various intensities (0.2 W/cm^2^, 0.5 W/cm^2^, 0.7 W/cm^2^, 1.0 W/cm^2^) to evaluate the healing properties of the nanofiber mat and establish the relationship between NIR and light intensity. Simultaneously, to evaluate the photothermal stability of the microgel composite fiber membrane, nanofibers were subjected to a power of 1.0 W/cm^2^, leading to a temperature rise to around 52 °C. Upon laser deactivation, the nanofibers exhibited the capability to revert to ambient temperature. This cycle of laser activation and deactivation was replicated thrice. The optically coupled infrared semiconductor laser (LR-MFJ-808/5000 mW) utilized was sourced from Larry Optoelectronics Technology Co., Ltd. (Changchun, China), while the thermal imaging temperature detector (UTi320E) was obtained from Uni-Trend Technology Co., Ltd. (Dongguan, China).

The determination of photothermal conversion efficiency (η) of MGACS nanofiber mat was carried out as following Eqs. [35, 36].

1 $$\eta =\frac{\text{h}\text{S}\left({\text{T}}_{\text{m}}-{T}_{r}\right)-{Q}_{0}}{\text{I}\left(1-{10}^{-{A}_{808}}\right)}$$

2 $${\tau }_{s}=\frac{{\text{m}}_{\text{d}} {C}_{d}}{\text{h}S}$$

3 $${Q}_{0}=hS({\text{T}}_{\text{m}, \text{w}}-{T}_{r})$$

4 $$t=-{\tau }_{s}\text{ln}\left(\frac{{\text{T}}_{\text{c}}-{\text{T}}_{\text{r}}}{{\text{T}}_{\text{m}}-{\text{T}}_{\text{r}}}\right)$$

Where η represents the heat transfer coefficient, while S signifies the surface area of the nanofiber mat. T_m_ and T_r_ denote the maximum temperature and room temperature of photothermal conversion materials, respectively. Q_0_ was identified using pure water as a reference. I denotes the irradiation intensity. The value of hS can be determined based on Eq. (2). The A_808_ signifies the 808 nm absorbance of MGACS nanofiber mat. The mass and specific heat capacity of the substance under test are represented by M_d_ (1 g) and C_d_ (4.2 J/(g⋅℃)), respectively. The real-time temperature during the cooling period, as captured by the thermal imager, is referred to as T_c_ And T_m, w_ is the maximum temperature reached by pure water under NIR irradiation.

Concurrently, both pure MXene and microgel-encapsulated MXene were submerged in 0.5 ml PBS and exposed to daily NIR irradiation for 5 min each day over 7 consecutive days. This experimental configuration sought to examine the dynamic correlation between temperature increment and time over successive days, offering a thorough evaluation of the photothermal heating stability of microgel-encapsulated MXene.

#### Thermogravimetric analysis

Following the fabrication of the nanofiber membrane, the moisture content is eliminated by employing a freeze dryer. Subsequently, the samples underwent thermogravimetric analysis using a thermogravimetric analyzer (Mettler TGA/DSC3+, Delaware, USA) at a heating rate of 10 °C·min^− 1^ within the temperature range of 30–800 °C.

#### Cell viability and biocompatibility assay

L929 cells, up to the fifth passage, were utilized to evaluate the biocompatibility of the electrospun membranes. These cells were propagated in α- Dulbecco’s Modified Eagle Medium (α-DMEM) culture medium enriched with 10% fetal bovine serum, under conditions of 37 °C and 5% CO_2_. The culture medium was replenished every 48 h. For subsequent co-culture experiments relating to biomaterial interactions, cells were strategically positioned in the lower chamber of a Transwell insert (CORNING, China) with a pore size of 0.4 μm. Simultaneously, the nanofiber membrane was situated on the upper chamber of the same apparatus. Upon assembly, the entire device was incubated at conditions of 37 °C and 5% CO_2_.

The CCK-8 assay was employed to assess the viability of L929 cells when co-incubated with membrane samples [37]. L929 cells (1 × 10^4^) were co-incubated with nanofibers for a period of 3 h, 6 h, and 12 h under conditions of 37 °C and 5% CO_2_. The absorbance of the resultant supernatant was quantified using a microplate reader at 450 nm. For the CCK-8 assay, the cell viability was ascertained using the formula:

$$\eqalign{& {\rm{Cell}}\;{\rm{Viability}}\left( \% \right) \cr & \,\,\,\,\,\,\,\,\, = \left[ {{{\left( {{\rm{O}}{{\rm{D}}_{}}_{\rm{measured}} - {{\rm{OD}}{}}_{\rm{Blank}}} \right)} \over {\left( {{\rm{O}}{{\rm{D}}_{{\rm{Control}}}}{\rm{ - O}}{{\rm{D}}_{}}_{\rm{Blank}}} \right)}}} \right] \times 100\% \cr}$$

Within this framework, OD _blank_ corresponds to α-DMEM containing 10% fetal calf serum, while OD _control_ represents the spectral reading of the cell solution. Simultaneously, the OD _measured_ involves solutions of cell cultures bound to nanofiber membranes.

The viability of L929 cells was further explored using fluorescence-based assays for live/dead viability/cytotoxicity, by the established guidelines for coculturing with a range of materials. After a period of 1 and 3 days of co-incubated alongside the membranes, the cells were subject to staining, and subsequently incubated in a dark environment for a duration of 20 min. Post-staining, the samples underwent imaging using a Nikon ECLIPSE Ti2-E fluorescence microscope. Green fluorescence signified the presence of live cells, whereas the dead cells were indicated by red fluorescence. Cell counting was conducted using ImageJ software v1.53, and the resultant data served to determine cell viability through the designated formula.

$$\eqalign{& {\rm{Cell}}\;{\rm{Viability}}\left( \% \right) \cr & \,\,\,\,\,\,\,\,\, = \left[ {{{{{\rm{N}}_{}}_{\rm{Live}}} \over {\left( {{{\rm{N}}_{{\rm{Live}}}}{\rm{ + }}{{\rm{N}}{}}_{\rm{Dead}}} \right)}}} \right] \times 100\% \cr}$$

where N _live_ represents the number of live cells, and N _dead_ represents the number of dead cells.

#### Cell morphology assay

To evaluate cellular morphology, cells were subjected to staining using iFluor 488 phalloidin and DAPI. The samples underwent an incubation period with iFluor 488 phalloidin for 4 h at 37 °C, followed by a subsequent incubation period with DAPI for an additional 30 min. Post incubation, the samples were gently washed with PBS, a process that was repeated thrice. After the staining process was completed, the cells were imaged employing an ECLIPSE Ti2-E inverted fluorescence microscope (Nikon, Japan). The images thus procured were then analyzed utilizing ImageJ software v1.53.

#### Planktonic bacteria and biofilm preparation

Planktonic bacteria were prepared using S. aureus, MRSA, and E. coli strains. These were cultured in 4 mL of Luria-Bertani (LB) liquid medium and incubated at 37 °C with continuous agitation (200 rpm) overnight for downstream experiments. To prepare the mature biofilm, an 800 µL aliquot of the bacterial suspension (10^8^ cfu/mL) was added to a 24-well plate containing LB medium. The medium was replaced daily during the 2-day incubation period. Following this, the medium was discarded, and non-adherent bacteria were removed by washing with PBS three times.

#### Evaluation of antibacterial activity in vitro

To evaluate the antimicrobial efficiency of the nanofiber membranes in vitro, bacterial viability was ascertained through both conventional plate counting and live/dead staining methodologies. A milliliter of three bacterial strains S. aureus, MRSA, and E. coli were combined with various nanofiber pads and incubated for 12 h at 37 °C with persistent agitation. The group subjected to NIR treatment underwent additional 808 nm laser (1 W/cm^2^) irradiation for a period of 5 min. Initial assessment of colony forming units (CFU) post-treatment was executed utilizing the standard plate count (SPC) method. The resultant bacterial solution was diluted by a factor of 5.0 × 10^3^, and 20 µl of each solution was spread onto an agar plate. Following a 24-hour incubation period at 37 °C, CFU was enumerated and analyzed using ImageJ software (v1.53).

Concurrently, bacterial samples from each treated group were collected and rinsed with PBS buffer solution (pH 7.4), employing centrifugation at 3000 rpm. These samples were then marked with bacterial live/dead staining dye, followed by a reaction period of 15 min. Observations and imaging were subsequently carried out utilizing an inverted fluorescence microscope (ECLIPSE Ti2-E, Nikon, Japan).

#### Evaluation of biofilm dispersion ability in vitro

Biofilms, cultivated as per the aforementioned methodology, were segregated into different groups: the control group, CS group, GACS group, MGACS group, and MGACS + NIR group. The NIR group was uniquely subjected to illumination at a wavelength of 808 nm and 1 W/cm^2^ for 5 min. Post-treatment, the biofilm biomass was quantitatively evaluated using crystal violet staining. As per the treatment regimen, the culture medium was initially discarded, followed by a wash with PBS. The residual biofilm was subsequently stabilized with absolute ethanol and stained with a crystal violet staining solution. Post a 10-minute incubation period, the surplus dye solution was eliminated, and the biofilm was dissolved again using 30% glacial acetic acid. The absorbance of the resultant eluate was measured at 590 nm using a microplate reader.

Moreover, the eradication of the biofilm was validated using SEM. Biofilms were propagated on sterile titanium slides, exposed to diverse treatments, fixed with paraformaldehyde for a 12-hour duration at 4 °C, and subsequently dehydrated using a gradient of ethanol concentrations (50%, 70%, 80%, 90%, 95%, and 100%). Following the process of freeze-drying and gold sputtering, the samples were examined under the SEM.

To examine the three-dimensional architecture of the biofilm, biofilm cultivation was carried out using a 48-well plate. Following different treatments, the matured biofilms were rinsed and stained with the LIVE/DEAD BacLight Bacterial Viability Kit for a 15-minute duration in the absence of light. After this, the biofilm structure was scrutinized employing a fluorescence-inverted microscope (ECLIPSE Ti2-E, Nikon, Japan).

Following this, to further evaluate the bactericidal influence of PTT on bacteria residing within the biofilm post-biofilm disruption, 1 ml of LB medium was introduced to the treated biofilms in each of the previously mentioned groups. The biofilms were then incubated with continuous agitation at 37 °C for a period of 12 h. The CFU after the treatment was assessed employing the SPC methodology as detailed above.

#### In vitro evaluation of the mechanism of mild MPTT

To evaluate the effect of MPTT on cellular proliferation, the CCK-8 assay was utilized. Specifically, L929 cells were allotted into 24-well plates (1 × 10^4^ cells per well) for adhesion and propagated using DMEM (10% FBS) for 24 h. Following this, nanofiber membranes from each group were co-cultivated with the cells. The group labeled MGACS + NIR was subjected to NIR (0.5 W/cm^2^) radiation for 15 min daily for a consecutive five-day period (40–42 °C). On days 1, 3, and 5, a microplate reader was employed to detect the radiation at a wavelength of 450 nm and quantify the absorbance of the supernatant. Subsequently, cellular viability was determined employing the CCK-8 cell viability calculation formula as mentioned earlier.

To examine the influence of MPTT on cellular migration, artificial wounds were created by making longitudinal scratches when L929 cells reached approximately 90% confluence. More specifically, the MGACS group was exposed to NIR light radiation at an intensity of 0.5 W/cm^2^ to maintain cells within a temperature bracket of 40–42 °C for 15 min. Following this, the cells were immediately relocated to a cell culture incubator preset at 37 °C. The evaluation of cellular migration was carried out at specific intervals, namely 0, 6, and 12 h post-co-incubated, using an inverted phase contrast microscope (ECLIPSE Ti2-E, Nikon, Japan).

Angiogenesis assays were conducted to assess the capacity of MPTT in promoting blood vessel formation. In prechilled 24-well plates, 300 µl of Matrigel substrate was dispensed and left at 37 °C for 30 min to facilitate solidification. Human umbilical vein endothelial cells (HUVEC) were prepared at a concentration of 1 × 10^5^ cells/ml in a complete culture medium. Each well was supplemented with 300 µl of the cell suspension, corresponding to approximately 3 × 10^4^ cells, in conjunction with nanofiber membrane samples. The MGACS group underwent NIR irradiation (0.5 W/cm^2^) for 15 min while maintaining a temperature range of 40–42 °C. Following irradiation, the plates were promptly transferred to a 37 °C cell culture incubator. After a 6-hour incubation period, the formation of tubular structures was observed and recorded using an inverted phase contrast microscope (ECLIPSE Ti2-E, Nikon, Japan). Subsequent quantitative analysis of all captured image data was conducted using ImageJ software (v1.53).

We analyzed the effect of moderate thermal stimulation on the intracellular heat shock protein 47 (HSP47) content at the molecular mechanism level. L929 cells were allocated into 12-well plates with an approximate density of 2000 cells per well. Following this, they were subjected to moderate thermal stimulation for 15 min daily for a consecutive five-day period, adhering to the protocol outlined in prior studies. The cells were affixed to the plates using 4% formaldehyde, and subsequent permeabilization of cells was accomplished using PBS containing 0.1% Triton X-100— a pivotal step to facilitate the antibody penetration into cells. Post-permeabilization, cells were subjected to blocking using PBS containing 5% bovine serum albumin to deter nonspecific antibody binding. After blocking, cells were incubated with the primary antibody (goat anti-rabbit IgG 594) overnight at 4 °C. Post-incubation, cells were washed thrice with PBS to remove any unbound primary antibody. Subsequent incubation with a fluorescently marked secondary antibody (HSP47 monoclonal antibody) ensued for 1–2 h at room temperature. Following this incubation, cells were washed an additional three times with PBS to remove any unbound secondary antibody. Finally, DAPI was utilized for nucleic acid staining. The documentation of results was facilitated by capturing images using a fluorescent inverted microscope (ECLIPSE Ti2-E, Nikon, Japan), enabling the subsequent calculation of HSP47 fluorescence intensity.

#### Preparation of diabetic SD rats

All animal experiment studies were approved by the animal ethics committee of Nanfang Hospital of Southern Medical College (R202009.05, Guangzhou, China) and conducted in accordance with the National Research Council’s Guide for the Care and Use of Laboratory Animals. The experiment involved twenty-five male Sprague-Dawley (SD) rats, each weighing approximately 200 g, from the Experimental Animal Center of Southern Medical University. The rats were divided into five groups: control group, CS group, GACS group, MGACS group, and MGACS + NIR group. A type 1 diabetes rat model was established by intraperitoneal injection of streptozotocin (obtained from Shanghai McLean Biochemical Co., Ltd., Shanghai, China) in SD rats. The dosage ranged between 50 and 60 mg/kg body weight, in line with the established protocol. The rats’ blood glucose concentrations were monitored on day 0, day 3, day 6, and day 7 respectively. Blood samples were drawn from the tail vein for blood glucose testing (using Bayer Contour Next EZ, Leverkusen, Germany). Rats with blood glucose levels over 11.1 mmol/L were considered diabetic.

#### Animals and surgical procedures

Twenty-five Sprague-Dawley male rats, each weighing approximately 200 g, were utilized as experimental subjects and divided into five groups: control, CS, GACS, MGACS, and MGACS + NIR. The rats, sourced from the Experimental Animal Center of Southern Medical University, were anesthetized through intraperitoneal injection of 3% pentobarbital (100 µl/100 g body weight). Following hair removal and disinfection, a full-thickness circular skin defect with a diameter of 1 cm was created on the back of each rat. Subsequently, a suspension of Staphylococcus aureus (1 × 10^8^ CFU/mL) amounting to 20 µl was applied to the skin-deficient area to induce infection. The wounds were covered with gauze for a period of 6–8 hours to ensure successful infection establishment. Following this, CS, GACS, and MGACS film dressings were applied to the infected wounds.

In the initial 1–3 days following surgery, NIR irradiation (1 W/cm^2^) was applied to the rat skin wounds dressed with MGACS nanofiber membranes, maintaining the wound temperature between 50 and 52 °C for a duration of 5 min. Additionally, bacteria from the wound were harvested post-infection establishment and subsequent wound treatment, evaluating the antibacterial impact through the SPC methodology. Specifically, the collection of bacteria from wounds is accomplished via the following steps: Wound exudates were sampled post-infection and throughout the subsequent wound treatment process utilizing sterile, disposable throat swabs. Following this, the collection rod was positioned into a 15 ml centrifuge tube. The tubes were then filled with Luria-Bertani (LB) liquid medium and incubated overnight at a temperature of 37 °C. Further, from days 4–8 post-surgery, NIR (0.5 W/cm^2^) was employed to irradiate the rat skin wounds dressed with MGACS nanofiber membranes, sustaining them at a temperature of 40–42 °C for a period of 15 min. The progressive healing of the wounds was monitored during this timeframe. For the quantification of wound area, we procured digital images on days 1, 3, 5, 7, and 14 post-surgery and utilized ImageJ software for the calculation of the wound area. The percentage of wound healing size was computed using the following formula: [(A_0_ – A _(1, 3, 5, 7, 14)_ / A_0_] x 100%, where A_0_ denotes the wound area on day 0, and A _(1, 3, 5, 7, 14)_ represents the wound area on days 1, 3, 5, 7, and 14 respectively.

Immunohistochemical examination of the wound area was carried out by procuring tissue samples on day 14. The harvested tissue was preserved in 4% paraformaldehyde overnight before being embedded in paraffin. Sections were subsequently prepared for staining with hematoxylin and eosin (H&E), Masson’s trichrome, CD31(ab182981, 1: 2000), VEGF(ab315238, 1:2000), and collagen types I(ab270993, 1:2000) and III(ab184993, 1:2000). Observations of the tissue samples were conducted using a light microscope.

#### Statistical analysis

All in vitro studies were repeated three times. The resulting data were subjected to a one-way analysis of variance (ANOVA), followed by a Bonferroni post hoc test for statistical analysis, using GraphPad Prism 8.0 software. The error bars denote the mean ± standard deviation (SD) of the obtained values. (*P < 0.05, **P < 0.01, and ***P < 0.001).

## Results and discussion

### Nanofiber fabrication and characterization

#### SEM scan of the nanofiber membranes

**Fig. 2:**
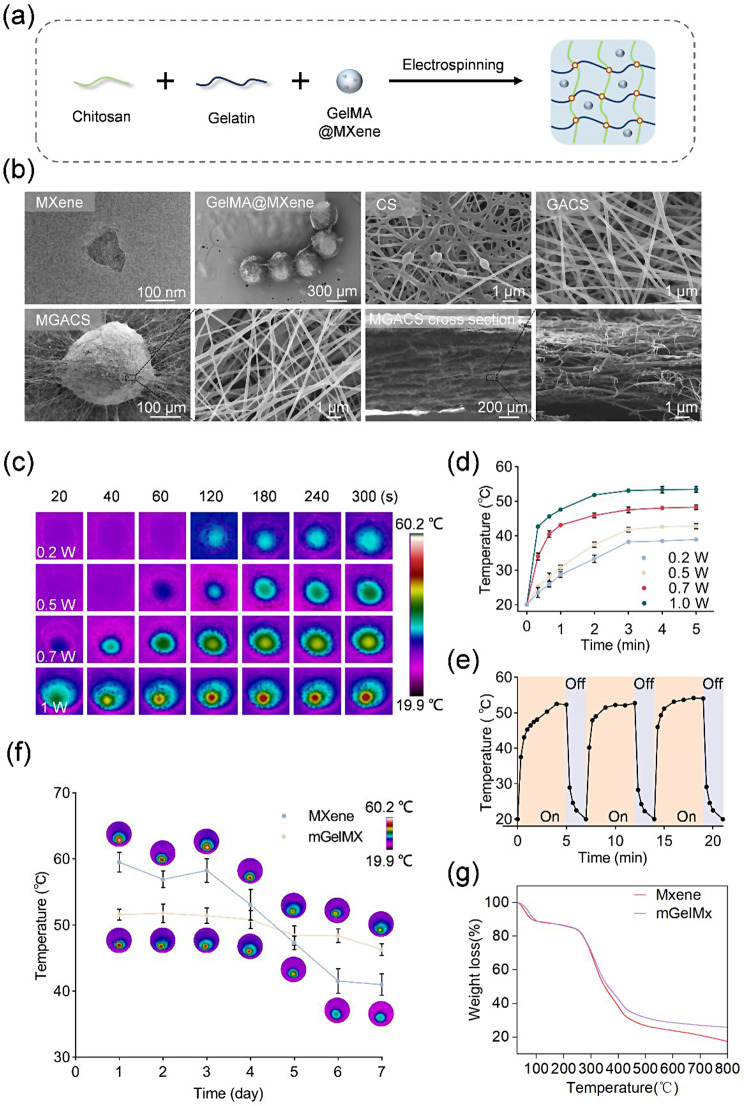
Characterization of MXene microgel-loaded nanofiber membranes: (**a**) Concise depiction of the fabrication process for MXene microgel-loaded nanofiber membranes. (**b**) TEM images of Mxene, SEM images of GelMA@Mxene, CS, GACS, and MGACS nanofiber membranes, inclusive of cross-sectional SEM images of MGACS nanofiber membranes. (**c**-**d**) Thermographic analysis and photothermal properties of nanofiber membranes, prepared from a 50 µg/ml MGACS mixed solution, under varying NIR power conditions. (**e**) Photothermal heating/cooling curves of nanofiber membranes (50 µg/mL) after three cycles of 808 nm laser irradiation (1 W/cm^2^). (**f**) Examination of photothermal properties of Mxene and GelMA@Mxene under continuous NIR irradiation. (**g**) Thermal degradation stability of Mxene and GelMA@Mxene.

Figure 2a delineates the manufacturing process of mGelMx-loaded nanofiber membranes. SEM and TEM images depict the original state of Mxene nanosheets and their state after 12 h of sonication. The resulting Mxene consistently displays an accordion-like structure with an average thickness of 1.5 μm and lateral dimensions of roughly 4.75 ± 0.78 μm (Fig. S1a-b). After 12 h of ultrasonic treatment, a thin structure is procured with a lateral dimension of approximately 184.93 ± 50.85 nm (Fig. 2b & Fig. S1c). Meanwhile, SEM was utilized to examine the morphology of the resulting nanofibers and the fiber diameter of each sample was measured (Fig. 2b). As shown in Fig. S2a, SEM images demonstrate the effective transformation of the GACS mixed solution into nanofibers. Interestingly, compared to pure CS nanofibers, GACS nanofibers show an increase in smoothness and uniformity as the GA content escalates. The fiber diameter of CS is 242.96 ± 53.5 nm, and that of GACS is 228.32 ± 42.77 nm (Fig. S2b-d), highlighting the significant improvement in CS electrospinning performance facilitated by GA.

Additionally, SEM observations indicated that the photothermal microspheres were spherical with a diameter of 369.73 ± 61.99 μm (Fig. S1d). Electrospun interconnected filamentous structures skillfully encapsulate GelMA microspheres. The inclusion of MXene microspheres further reduces the average diameter of the nanofibers to 214.16 ± 69.84 nm (Fig. S2d), potentially due to the increased charge density of the spinning solution resulting from its polar groups. Consequently, enhanced electrostatic forces act on the spinning solution, leading to a more noticeable stretching of the charged liquid into finer fibers. It is a well-accepted notion that thinner fibers provide more adhesion sites for cells, thereby fostering improved cell proliferation and signaling [38]. Moreover, SEM analysis of the cross-sectional thickness of the nanofiber membrane showed a thickness of 889.23 ± 71.87 μm.

#### Spectral analysis via FT-IR

The FTIR spectra of CS, GACS, and MGACS fiber membranes are displayed in Fig. S3d, exhibiting analogous absorption peaks. The absorption bands spanning from 1200 to 900 cm^− 1^ correspond to the saccharide structure of CS and are apparent in all specimens. Additionally, NH and OH bending peaks (3600 –3100 cm^− 1^) are present across all spectra. For the CS spectrum, the peaks at Amide I (C = O) (1645 cm^− 1^) and Amide II (1565 cm^− 1^) are relatively faint, likely due to the high deacetylation degree of CS. In the GACS spectrum, characteristic absorption bands of GA are observed at 1655 cm^− 1^, 1549 cm^− 1^, and 1338 cm^− 1^, corresponding to N-H bending vibrations of Amide I, II, and III, respectively [39]. Moreover, the vanishing of the amino vibration peak at 1565 cm^− 1^ and the distinct peak at 1655 cm^− 1^ following the polymerization of the GA and CS mixture suggest potential amidation reactions between carboxyl and amino groups. Furthermore, in the GACS membrane spectrum, the intensity of peaks at 1200 –900 cm^− 1^ lessens, possibly indicative of hydrogen bonding interactions occurring within the GACS mixture. Upon the integration of mGelMx into the GACS nanofiber membrane, FTIR presents similar absorption peaks without triggering the formation of any chemical bonds.

#### Swelling, degradation properties, and water contact angle

As illustrated in Fig. S3c, f, g, the incorporation of GA and mGelMX results in nanofiber membranes with smaller water contact angles and remarkable swelling performance. Concurrently, the degradation of the electrospun nanofiber membrane markedly escalates upon the incorporation of GA, culminating in nearly total degradation by day 14 (Fig. S3e). This observation suggests that the addition of GA leads to a modest increase in the swelling capacity owing to its properties that inhibit crystallization, potentially contributing to a decrease in chitosan’s crystallinity [40]. Hydrophilic dressings with a higher swelling ratio offer advantages in absorbing exudate from infected wounds and removing harmful substances from the wound [41]. Moreover, the nanofiber mat with added GA undergoes almost complete degradation by 14 days, which perfectly matches the healing speed of the defective skin and obviates the need for changing wound dressings in clinical applications [42].

#### Photothermal activity of nanofiber membranes

We examined the photothermal behavior of mGelMx nanofiber thin films of varying concentrations under NIR radiation at 1 W/cm^2^ by tracking temperature fluctuations. Remarkably, the film with a concentration of 50 µg/ml in 0.5 ml deionized water rose swiftly to approximately 52 °C within 2 minutes and reached a stable plateau around 52 °C (Fig. S3a-b). In contrast, films with concentrations of 20 µg/ml and 40 µg/ml attained 42 °C and 49 °C respectively in 2 minutes under the identical irradiation power, while the film with a 60 µg/ml concentration achieved 57 °C after 4 min and continued to escalate. Based on prior research, a temperature above 50 °C is necessary for effective bacterial inactivation via PTT, and temperatures surpassing 53 °C can induce rapid widespread necrosis of normal tissues. Therefore, the 50 µg/ml mGelMx nanofiber thin film was chosen for subsequent antibacterial experiments under 1 W/cm^2^ NIR power. Concurrently, we delved further into the heating performance of the 50 µg/ml MGACS film by altering the power of the NIR light. Observations revealed that at 0.5 W/cm^2^ radiation, the MGACS film could attain a temperature exceeding 40 °C within 3 min and stabilize at a plateau around 42 °C (Fig. 2c-d). Previous research indicated that thermal treatment at the range of 40–41 °C triggers endothelial cell tube formation and augments angiogenesis in a hindlimb ischemia mouse model. Thus, the 50 µg/ml MGACS nanofiber thin film was chosen for subsequent pro-healing experiments employing MPTT under 0.5 W/cm^2^ NIR power. Moreover, the nanofiber membrane demonstrated exceptional photothermal stability throughout three cycles of NIR light switching (Fig. 2e). The nanofiber membrane of MGACS demonstrates a photothermal conversion efficiency calculated at 37.15% (Fig. S5). This efficiency surpasses that of conventional photothermal agents like Au nanorods and Bi nanoparticles.

The above results proved that the MGACS nanofiber membrane has excellent sensitivity and controllability of photothermal heating performance. When exposed to NIR at 1 W/cm^2^, the temperature of the membrane quickly rises to around 52 °C. The superior performance might be attributed to its excellent photothermal conversion efficiency and high light absorption capacity, which is extremely beneficial for killing invading bacteria [43]. However, continued exposure to such temperatures can cause irreversible damage to surrounding skin tissue [44]. To avoid this collateral damage, NIR radiation is applied intermittently. This modulation application heats the membrane to a safety threshold of around 52 °C and then begins to cool. Then, by reducing the irradiation power to 0.5 W/cm^2^, and the temperature could be maintained at around 42 °C, which was ideal for conducting MPTT therapy.

#### Thermogravimetric analysis and photothermal stability

Figure 2g displays the outcomes of the thermogravimetric analysis (TGA) for pristine MXene and photothermal microspheres. MXene reveals an initial weight reduction commencing around 69 °C, while weight loss in photothermal microspheres begins around 76 °C. In addition, the TGA curve of MXene indicates inferior thermal stability compared to the photothermal microspheres. Particularly, within the temperature range of 60–300 °C, the weight reduction of pristine MXene is more rapid than that of the microspheres encapsulating MXene. A secondary degradation is detected at 310 °C, where MXene degrades more swiftly than the mGelMx microspheres as the temperature ascends. Moreover, we discovered that MXene encapsulated within microgels demonstrated enhanced photothermal stability under continuous NIR irradiation over a period of 7 days compared to pure MXene (Fig. 2f).

In conclusion, these data underscore that encapsulating MXene with GelMA microgels can serve as an exemplary PTA for programmable photothermal therapies, including both PTT and MPTT.

### In vitro antimicrobial activity of the nanofiber

**Fig. 3:**
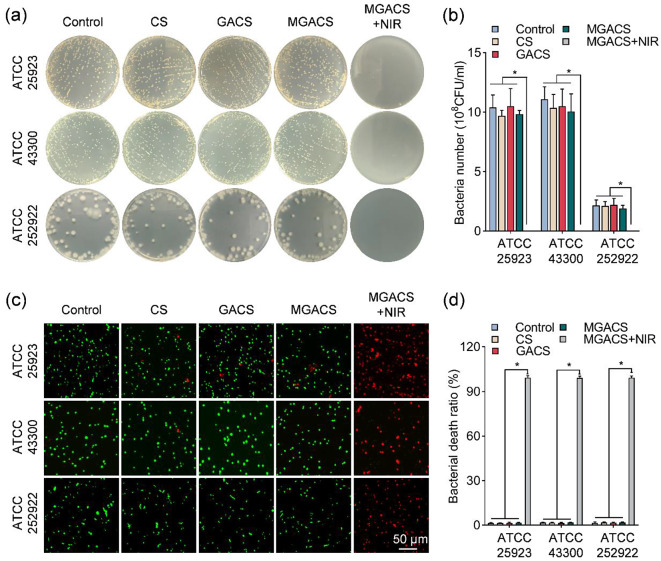
In vitro, Antibacterial efficacy of MXene microgel-loaded nanofiber membranes: (**a**-**b**) Utilization of the Colony Forming Unit (CFU) counting method to evaluate the in vitro antibacterial activity of MXene microgel-loaded nanofiber membranes, alongside a quantitative analysis of CFU counts. (**c**-**d**) Post-treatment assessment of bacterial viability for multiple strains (MSSA, MRSA, E. coli) and subsequent quantification of viable bacterial ratios using MXene microgel-loaded nanofiber membranes. (*n* = 3, *P < 0.05)

Figure 3a-b presents the survival rate of bacteria for each group of nanofiber membranes post-bacterial liquid treatment. After 12 h of co-culture, followed by photothermal treatment in the NIR group, nearly all bacteria in the MGACS + NIR group were eradicated. Compared to the control group and the group without NIR irradiation, there was a significant reduction in the bacterial survival rate. These observations are corroborated by the bacterial live/dead staining (Fig. 3c-d). It is noteworthy that the MGACS nanofiber membrane demonstrated a wide spectrum and rapid bactericidal performance against both S. aureus, MRSA, and E. coli. All three tested bacterial strains were completely eradicated within 5 min, which is much faster than traditional antibiotics (usually hours) and previously reported PPT (30 min) [45].

### Antibiofilm properties of the nanofiber in vitro

**Fig. 4:**
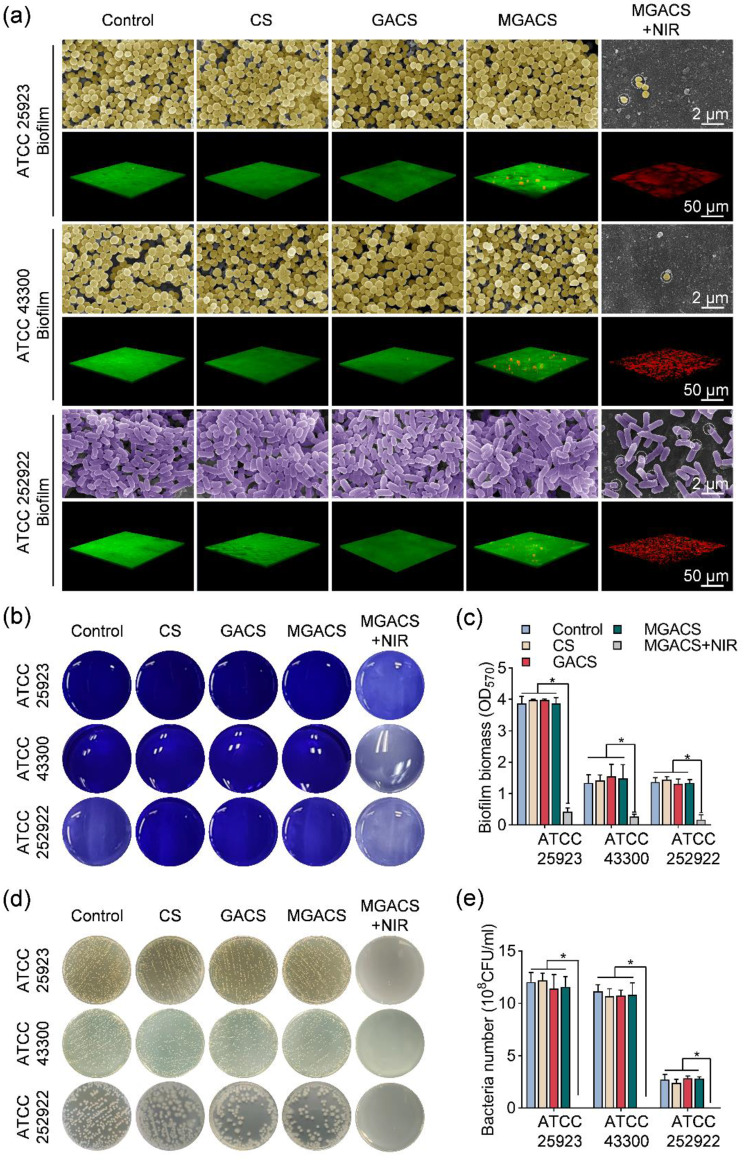
In vitro anti-biofilm efficacy of MXene microgel-loaded nanofiber membranes: (**a**) Z-stack scan of live/dead staining and SEM images of biofilms generated by three types of bacteria (MSSA, MRSA, and E. coli) following treatment with MXene microgel-loaded nanofiber membranes. (**b**-**c**) Visualization of biofilm formation using crystal violet staining for MSSA, MRSA, and E. coli after various treatments, along with measurements of biofilm biomass. (**d**-**e**) Representative images of SPC and corresponding CFU counts for MSSA, MRSA, and E. coli following different treatments. (*n* = 3, *P < 0.05)

Z-stack scans from fluorescence microscopy and SEM indicated that the biofilms in the control, CS, GACS, and MGACS-treated groups remained largely viable and structurally unscathed. Conversely, the MGACS + NIR group disrupted the compact biofilm, leading to a significant loss of biomass (Fig. 4a). The outcomes of crystal violet staining disclosed that without the introduction of NIR, all nanofiber membranes demonstrated a weak capacity to eradicate biofilms. However, with the inclusion of NIR, the biofilm elimination capability of the MGACS group markedly improved (Fig. 4b-c). Importantly, following the disruption of the biofilm by NIR treatment, a remarkable bactericidal effect against the bacteria within the biofilm was observed upon its dissolution. These findings affirm the efficient eradication and inhibitory impacts of PTT on biofilms.

In addition, the respective biofilm was also successfully disintegrated within 10 min of NIR treatment, and the embedded bacteria were eliminated, compared to a 20-minute therapy reported previously (Fig. 4d-e). The excellent bactericidal performance could be explained by the antibacterial mechanism of PTT [46]. Firstly, increased temperatures cause a rise in the temperature of bacteria and cells within the biofilm. This thermal effect can lead to cell membrane rupture and protein denaturation, compromising the biofilm’s structural integrity [47]. Secondly, the elevated temperature triggers a rapid reduction in cell vitality within biofilms, resulting in swift cell lysis. This occurrence can be ascribed to the combination of cell membrane rupture and damage to the internal cellular structure caused by high temperatures [48]. Simultaneously, heightened temperatures disrupt interactions between bacteria and cells within biofilms, including cell-to-cell adhesion and signaling. This disruption potentially aids in biofilm disintegration and the dispersion of individual cells [49]. Collectively, these mechanisms work in synergy to induce biofilm rupture and cell death.

### Cell viability and biocompatibility assay

**Fig. 5:**
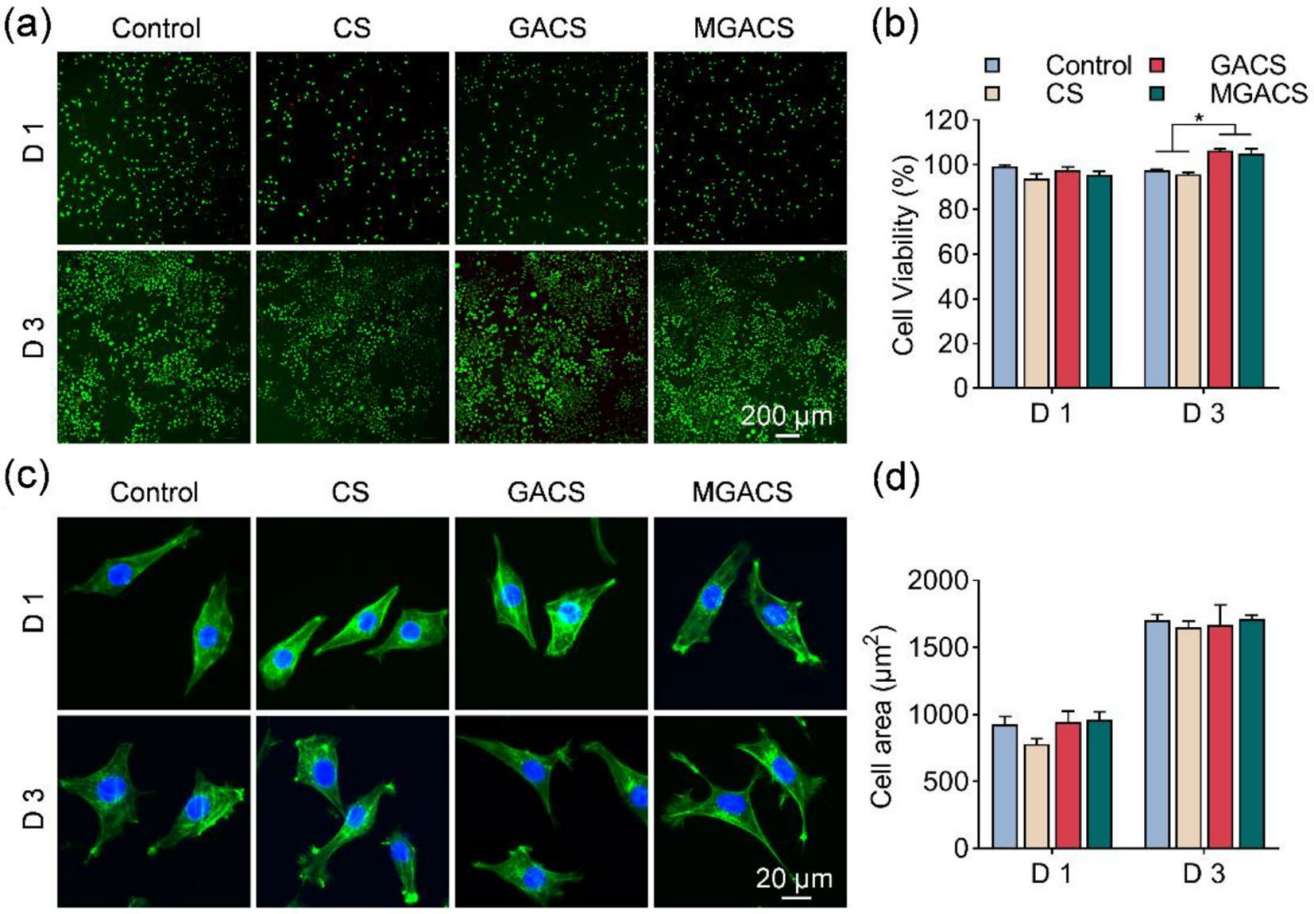
Cytotoxicity analysis of MXene microgel-loaded nanofiber membranes: (**a**, **b**) The viability of L929 cells in the presence of MXene microgel-loaded nanofiber membranes was evaluated via live/dead staining, where green signifies live cells and red signifies dead cells. (**c**, **d**) Visualization of L929 cell spreading post-treatment with MXene microgel-loaded nanofiber membranes using phalloidin (green signifies actin filaments) and DAPI (blue signifies nuclei) staining. (*n* = 3, *p < 0.05)

Despite certain studies suggesting that MXene could induce oxidative stress in cells, thereby leading to cell damage or death and raising potential safety concerns, the findings presented in Fig. S4a indicate that the biological functionality of L929 fibroblasts remains predominantly unaffected when the MXene concentration is decreased to 50 µg/ml. The encapsulation of MXene within GelMA microspheres augments its stability, further mitigating concerns related to the biosafety of the membrane. This is evidenced by the absence of any discernible adverse impact on cell proliferation and spreading behavior (Fig. 5). Notably, L929 cells co-cultured with GA -GA-supplemented membranes demonstrated an enhanced cell proliferation activity compared to CS membranes. This can be attributed to GA’s unique cell-binding sites, and its ability to enhance the hydrophilicity of the nanofibers, thus promoting cell attachment and proliferation [50].

### In vitro evaluation of the mechanism of MPTT

**Fig. 6:**
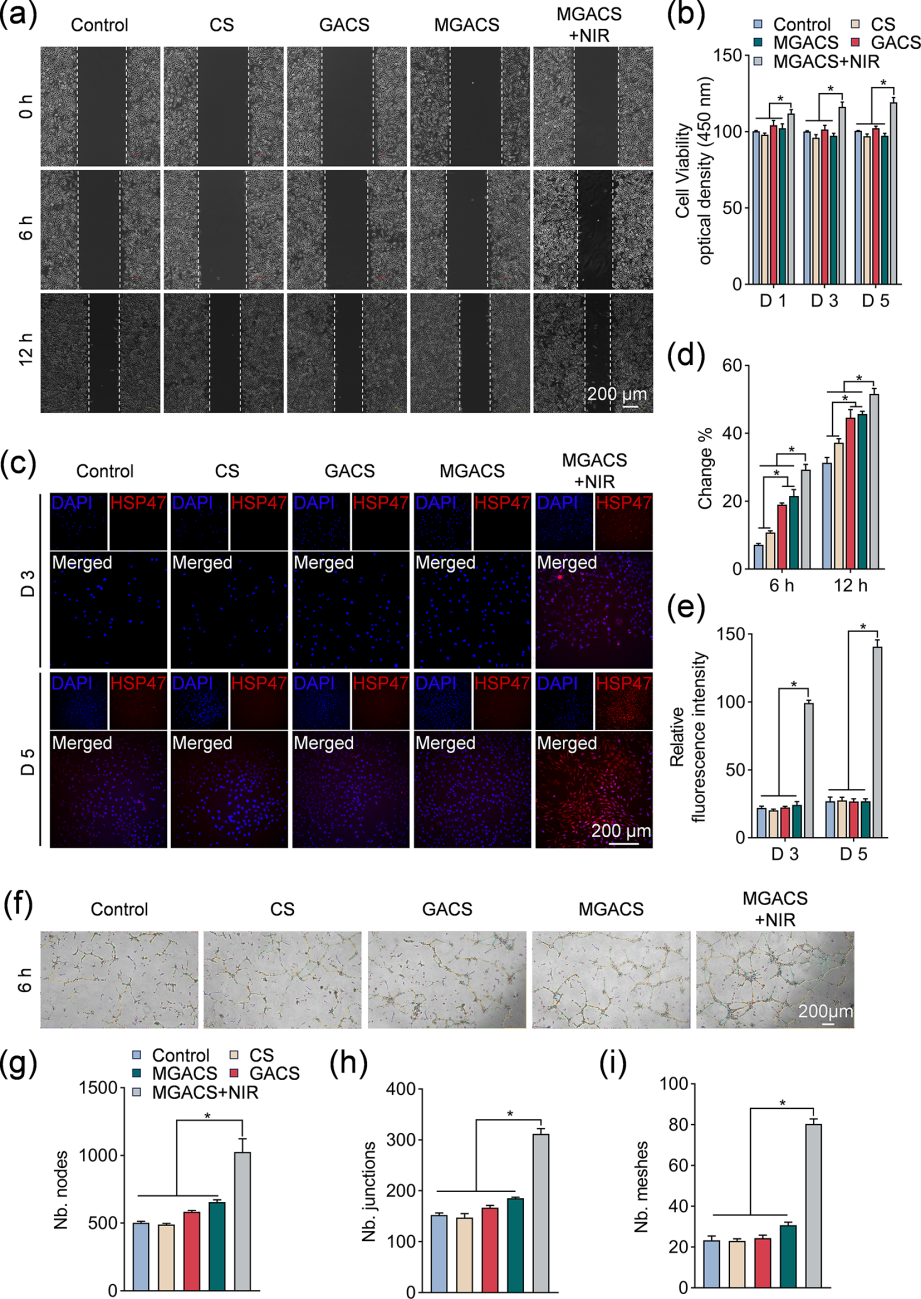
Effects of MPTT (40–42 ℃) on cell migration and angiogenesis in vitro: (**a**, **d**) The results of an L929 cell scratch test and analysis of migration rates following mild photothermal treatment (12 h, BF bright field). (**b**) A CCK-8 assay of the cell viability of L929 cells post MPTT. (**c**, **e**) Immunofluorescence staining of HSP 47 from different groups, along with a quantitative analysis of relative fluorescence intensity in cells stained with HSP 47 is presented. (**f**) An image displays the tube formation of HUVECs on a Matrigel matrix (BF bright field, *n* = 3). (**g**-**i**) The tube-forming ability of HUVECs post-treatment with PCH-based nanofiber membranes is analyzed. (*n* = 3, *P < 0.05)

As shown in Fig. 6, the localized moderate thermal stimulation produced by MGACS under NIR light exposure enhanced fibroblast recruitment, accelerated cell differentiation, and boosted angiogenesis. Following a 15-minute exposure to NIR irradiation, the culture medium’s temperature increased to between 40 and 42 °C. After five days of continuous NIR stimulation, the relative cell viability of the MGACS + NIR group, in comparison to the control group, rose to 110% after the initial day and climbed to 120% after the third day. No significant variations were detected between groups not exposed to NIR and the control group (Fig. 6b). In the cell wound healing assays (Fig. 6a, d), L929 cells demonstrated migration into vacant spaces over a duration of time. Post 12 h, the MGACS + NIR treated group showed a superior migration rate (51.64 ± 0.88%) compared to the control group (31.32 ± 0.84%). This occurrence was ascribed to MPTT, which aids L929 cell migration and thereby contributes to wound repair. Additionally, the integration of GA also positively impacts cell migration.

To probe the impact of mild thermal stimulation at the molecular mechanism scale, we proceeded to evaluate the alterations in intracellular HSP47 expression post-MPTT treatment. After mild thermal stimulation (40–42 °C) for 15-minute daily intervals over five consecutive days, MGACS + NIR demonstrated the most intense red fluorescence, signifying the maximum expression of HSP47 (Fig. 6c, e). In contrast, the control group and the membrane devoid of NIR irradiation revealed no significant red fluorescence following 5 days of cell co-culture. These findings align with the outcomes of angiogenesis studies that investigated the impact of mild thermal stimulation on endothelial cell angiogenesis in an in vitro environment. Relative to the control group and membranes devoid of NIR irradiation, mGelMx-loaded nanofibrous membranes considerably augmented the formation of human umbilical vein endothelial cells (HUVEC) tubes following MPTT treatment (Fig. 6f-i).

HSP47, a heat shock protein involved in collagen production, may enhance the quality of the ECM, aiding in the creation of a sturdy structural scaffold necessary for efficient cell movement [51]. Moreover, the heightened expression of HSP47 could potentially stimulate signaling pathways associated with angiogenesis, particularly the transforming growth factor-β (TGF-β) signaling pathway [52]. These pathways can trigger the transformation of endothelial cells into vascular endothelial cells, thus promoting the generation of new blood vessels [53]. In conclusion, the MGACS nanofiber membrane exhibits remarkable proficiency in fostering cell migration and the formation of blood vessels, ultimately facilitating effective wound healing.

### Wound healing assessment in vivo

**Fig. 7:**
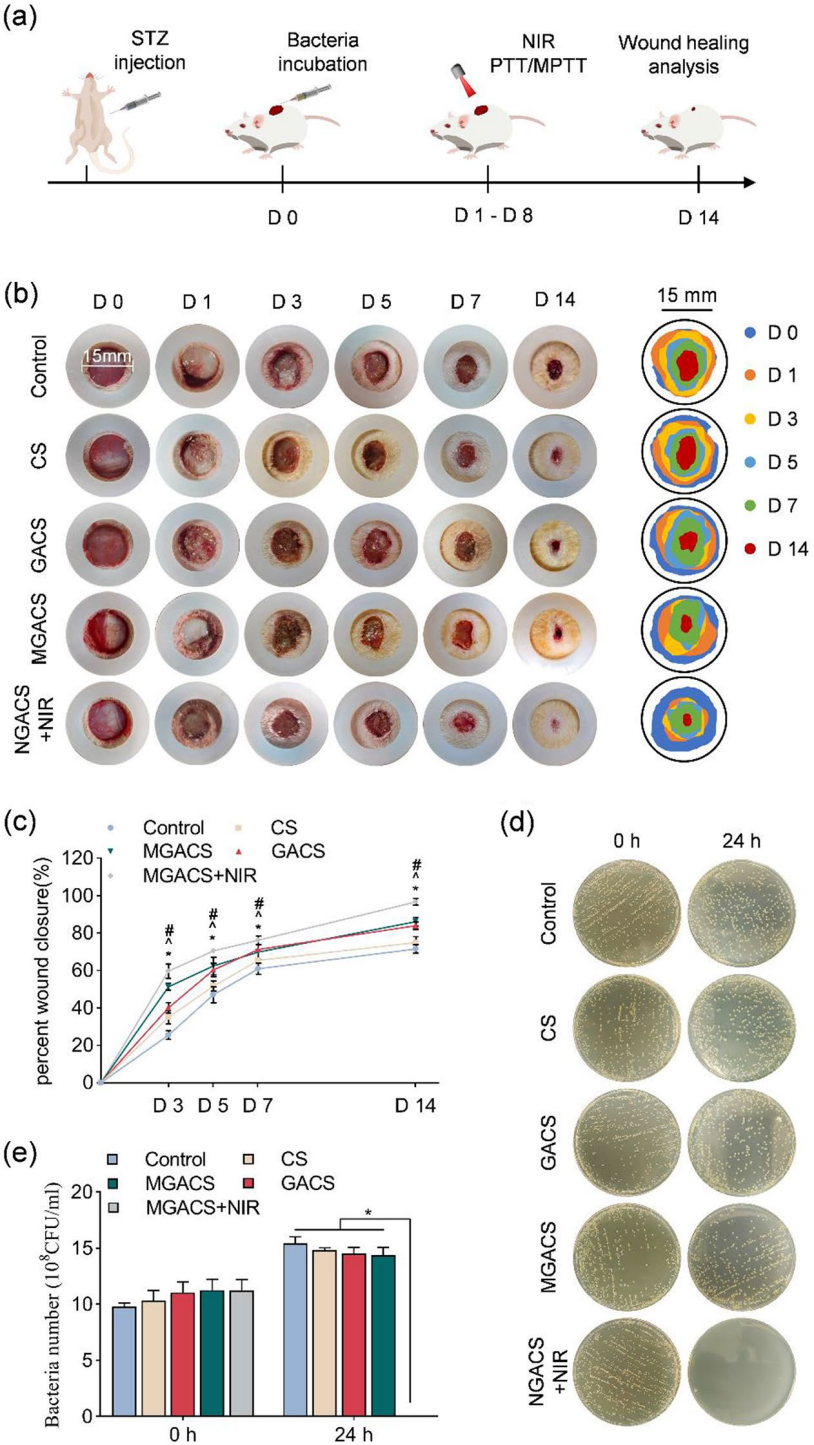
MXene microgel-loaded nanofiber membranes stimulated infected diabetic wound healing: (**a**) A schematic illustration of various stages of in vivo animal testing. (**b**) Images depict the dynamics of wound contraction at the wound site for the control, CS, GACS, MGACS, and MGACS + NIR groups over specific time frames, alongside a simulation of wound dynamics. (**c**) A quantitative analysis of the wound healing rate is provided. (**d**) Representative images of in vivo antimicrobial SPC and corresponding CFU. (*n* = 3, *P < 0.05)

The objective of this study was to scrutinize the effects of electrospun nanofiber dressings on the promotion of healing within a rat model of diabetic wound contamination. Diverse dressings were applied to these wounds: CS, GACS, MGACS, and MGACS + NIR, as indicated in Fig. 7.

The bacterial count in wounds, post-establishment of infection, and post-treatment with dressings in each group was conducted using SPC. As depicted in Fig. 7d-e, the group treated with MGACS + NIR dressing exhibited the most significant bactericidal effect, with the bacteria in PTT-treated wounds virtually eliminated. These findings powerfully underscore the superior antibacterial efficacy of PTT in vivo.

Images were captured, and the wound surface area was gauged using a plastic circle (15 mm inner diameter) as a benchmark to record wound healing progression. Amongst all groups, the MGACS + NIR dressing group displayed the most rapid wound healing rate and the most potent bactericidal effect. The comparison of skin defect area and wound healing rate is depicted in Fig. 7b-c. Two weeks post-surgery, the rats were euthanized for tissue sample collection.

**Fig. 8:**
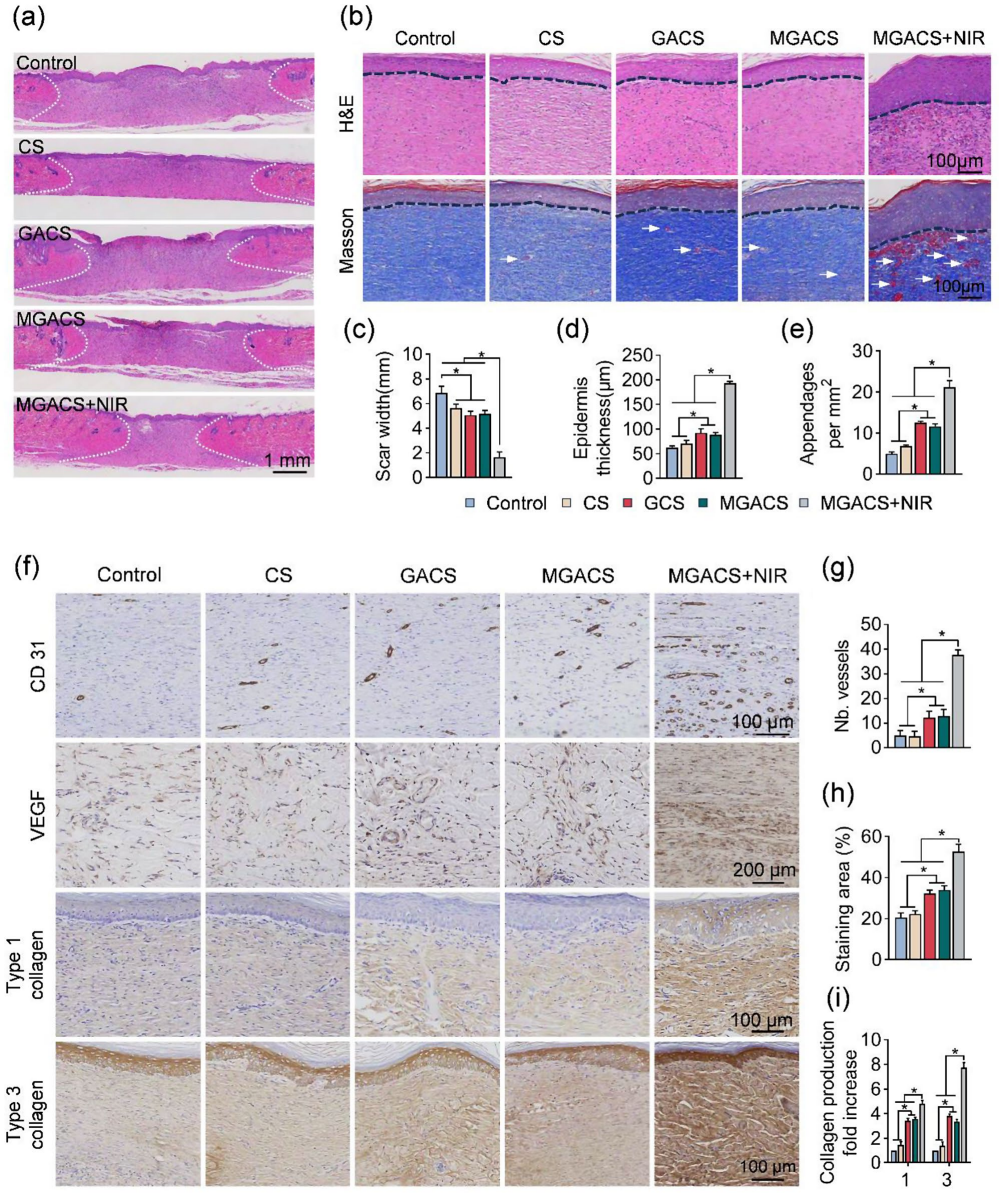
Assessment of wound repair via H&E, Masson, and immunohistochemical staining: (**a**) Healing of wounds is represented by H&E staining, with white dashed lines marking unhealed scars. (**b**) Images were stained with H&E and Masson, with dashed lines indicating the epidermis and arrows pointing towards blood vessels. (**c**) Scar widths for different groups are analyzed using ImageJ. (**d**) Analysis of epidermal thickness in wounds post nanofiber membrane treatment is carried out using ImageJ. (**e**) Quantification of blood vessels at wound locations is performed via ImageJ. (**f**) Images of staining for CD31, HIF-1α, type 1 collagen, and type 3 collagen in the control, CS, GACS, MGACS, and MGACS + NIR group. (**g**) The number of vessels based on CD31 staining images. (**h**) The staining area of VEGF. (**i**) Quantification of type I and type III collagen deposition. (*n* = 3, *P < 0.05)

To assess the quality of healed skin, histological analyses using H&E and Masson staining techniques were first employed, as illustrated in Fig. 8. Notably, wounds treated with MGACS nanofiber membrane and programmed PTT and MPTT exhibited minimal scar width, enhanced epidermal thickness, and more new blood vessels formation (Fig. 8a-e). Consequently, the electrospun fiber membrane antibacterial dressing has several notable benefits. On one hand, it effectively diminishes scar formation and narrows the scar width of the wound, thanks to its superior biological properties and structural design that enhance the wound healing process and curtail inflammatory reactions and scar formation. Additionally, the dressing bolsters epidermal thickness and improves the state of the skin surrounding the wound. The mild photothermal stimulation generated by NIR light irradiation fosters cell proliferation and regeneration, quickening the growth and repair of epidermal cells [54]. By augmenting epidermal thickness, the dressing assists in reestablishing the integrity and functionality of compromised skin. Most importantly, the dressing fosters the formation of additional new blood vessels, thereby ameliorating blood supply and oxygen delivery to the wound [55]. The genesis of new blood vessels delivers more nutrients and oxygen, thereby advancing the wound healing and repair process [56]. To further elucidate the mechanism underlying the promotion of angiogenesis, we conducted an immunohistochemical analysis to examine the expression of VEGF and CD31. CD31 functions as an indicator for vascular endothelial cells, while VEGF is intimately linked with angiogenesis [57]. As depicted in Fig. 8f-h, the MPTT treatment group demonstrated a significant upregulation in CD31 and VEGF. Typically, VEGF triggers downstream signaling pathways, including the PI3K-Akt and MAPK pathways, by interacting with its receptor (VEGFR) [58]. These pathways are complexly intertwined with processes such as cell multiplication, migration, and survival. In addition, Type I and Type III collagen, the primary constituents of the dermal ECM, are posited to play a crucial role in wound healing and remodeling [59]. Immunostaining results further corroborate the beneficial effects of MPTT (Fig. 8i).

In summary, bacterial infection and inadequate blood circulation present substantial obstacles in the management of diabetic wounds [60]. Biofunctional wound dressings have been extensively researched and developed aimed at improving the healing process of infected diabetic wounds. In this research, we utilized electrospinning to integrate MXene-encapsulated microgels and CS / GA polymers into a microgel/nanofiber membrane dressing. This innovative engineered membrane represents the first application to successfully incorporate programmed antibacterial and pro-angiogenic functions within the same electrospun functional dressing material. By applying a near-infrared (NIR) intensity of 1.0 W/cm^2^, the local wound temperature can be increased to 52 °C, thereby aiding in photothermal therapy (PTT) sterilization. Further, the local wound temperature can be modulated to a milder 40–42 °C by adjusting the NIR power to 0.5 W/cm^2^, thus facilitating mild photothermal therapy (MPTT). The initial application of PTT exhibited rapid bactericidal and biofilm disintegration capabilities, while the subsequent application of MPTT demonstrated restored angiogenesis. The programmed PTT and MPTT achieved rapid and high-quality healing of diabetic infection wounds.

## Conclusions

In the present study, MXene-encapsulated microgels and CS / GA polymers were electrospun into microgel/nanofiber membrane dressings to stimulate diabetic-infected wound healing., The incorporation of GA into CS has augmented the electrospinnability, degradability, and capacity to promote cell proliferation of the nanofiber membrane dressing. The microgel/nanofiber membrane could realize programmed PTT and MPTT under NIR radiation. This PTT and MPTT strategy enhanced diabetic wound healing by swiftly achieving broad-spectrum bactericidal effects and promoting vascularization. Notably, the preparation of this multifunctional electrospun dressing holds significant promise for stimulating the healing of diabetic wounds.

### Electronic supplementary material

Below is the link to the electronic supplementary material.

Supplementary Material 1

## Data Availability

The datasets used and/or analysed during the current study are available from the corresponding author on reasonable request.

## Abbreviations

PPT: Programmed photothermal therapy
MPTT: Mild photothermal therapy
ECM: Extracellular matrix
CS: Chitosan
GA: Gelatin
GelMA: Gelatin Methacryloyl
VEGF: Vascular endothelial growth factor
MMPs: Matrix metalloproteinases
NIR: Near-infrared
DAPI: 4’,6-diamidino-2-phenylindole
DMSO: Dimethylsulfoxide
HF: Hydrofluoric acid
GACS: Gelatin-chitosan
mGelMx: MXene-infused microgels
SEM: Scanning electron microscopy
TEM: Transmission electron microscope
FT: IR-Fourier transform infrared spectroscopy
α: DMEM-α-Dulbecco’s Modified Eagle Medium
CFU: Colony forming units
SPC: Standard plate count
HUVEC: Human umbilical vein endothelial cells
HSP47: Heat shock protein 47
SD: Sprague-Dawley
H&E: Hematoxylin and eosin
ANOVA: Analysis of variance
TGA: Thermogravimetric analysis
TGF-β: Transforming growth factor-β

## References

[1] Zhang K, Ma Y, Luo Y, Song Y, Xiong G, Ma Y, Sun X, Kan C. Metabolic diseases and healthy aging: identifying environmental and behavioral risk factors and promoting public health. Front Public Health 2023, 11. 10.3389/fpubh.2023.1253506 PMC10603303 37900047

[2] Khunti K, Chudasama YV, Gregg EW, Kamkuemah M, Misra S, Suls J, Venkateshmurthy NS, Valabhji J (2023). Diabetes and multiple long-term conditions: a review of our current Global Health Challenge. Diabetes Care.

[3] McDermott K, Fang M, Boulton AJ, Selvin E, Hicks CW (2023). Etiology, epidemiology, and disparities in the burden of diabetic foot ulcers. Diabetes Care.

[4] Bai Q, Han K, Dong K, Zheng C, Zhang Y, Long Q, Lu T. Potential applications of nanomaterials and technology for diabetic wound healing. Int J Nanomed 2020:9717–43. 10.2147/IJN.S276001 PMC7721306 33299313

[5] Maheswary T, Nurul AA, Fauzi MB (2021). The insights of microbes’ roles in wound healing: a comprehensive review. Pharmaceutics.

[6] Horton WB, Barrett EJ (2021). Microvascular dysfunction in diabetes mellitus and cardiometabolic disease. Endocr Rev.

[7] Zeng Q, Qi X, Shi G, Zhang M, Haick H (2022). Wound dressing: from nanomaterials to diagnostic dressings and healing evaluations. ACS Nano.

[8] Liang Y, Liang Y, Zhang H, Guo B (2022). Antibacterial biomaterials for skin wound dressing. Asian J Pharm Sci.

[9] Mirhaj M, Labbaf S, Tavakoli M, Seifalian A (2022). An overview on the recent advances in the treatment of infected wounds: antibacterial wound dressings. Macromol Biosci.

[10] Chen Y, Shafiq M, Liu M, Morsi Y, Mo X (2020). Advanced fabrication for electrospun three-dimensional nanofiber aerogels and scaffolds. Bioact Mater.

[11] Chen Y, Dong X, Shafiq M, Myles G, Radacsi N, Mo X (2022). Recent advancements on three-dimensional electrospun nanofiber scaffolds for tissue engineering. Adv Fiber Mater.

[12] Sharma A, Kaur I, Dheer D, Nagpal M, Kumar P, Venkatesh DN, Puri V, Singh I (2023). A propitious role of marine sourced polysaccharides: drug delivery and biomedical applications. Carbohydr Polym.

[13] Azmana M, Mahmood S, Hilles AR, Rahman A, Arifin MAB, Ahmed S (2021). A review on chitosan and Chitosan-based bionanocomposites: promising material for combatting global issues and its applications. Int J Biol Macromol.

[14] Xing J, Zhang M, Liu X, Wang C, Xu N, Xing D. Multi-material electrospinning: from methods to biomedical applications. Mater Today Bio 2023:100710. 10.1016/j.mtbio.2023.100710 PMC10401296 37545561

[15] Kolipaka T, Pandey G, Abraham N, Srinivasarao DA, Raghuvanshi RS, Rajinikanth PS, Tickoo V, Srivastava S. Stimuli-responsive polysaccharide-based smart hydrogels for diabetic wound healing: design aspects, preparation methods and regulatory perspectives. Carbohydr Polym 2023:121537. 10.1016/j.carbpol.2023.121537 37985111

[16] Huo J, Jia Q, Huang H, Zhang J, Li P, Dong X, Huang W (2021). Emerging photothermal-derived multimodal synergistic therapy in combating bacterial infections. Chem Soc Rev.

[17] Şen Karaman D, Ercan UK, Bakay E, Topaloğlu N, Rosenholm JM (2020). Evolving technologies and strategies for combating antibacterial resistance in the advent of the postantibiotic era. Adv Funct Mater.

[18] Blackman LD, Qu Y, Cass P, Locock KE (2021). Approaches for the inhibition and elimination of microbial biofilms using macromolecular agents. Chem Soc Rev.

[19] Makvandi P, Zarepour A, Zheng X, Agarwal T, Ghomi M, Sartorius R, Zare EN, Zarrabi A, Wu A, Maiti TK (2021). Non-spherical nanostructures in nanomedicine: from noble metal nanorods to transition metal dichalcogenide nanosheets. Appl Mater Today.

[20] Huang Z, Cui X, Li S, Wei J, Li P, Wang Y, Lee C (2020). Two-dimensional MXene-based materials for photothermal therapy. Nanophotonics.

[21] George SM, Kandasubramanian B (2020). Advancements in MXene-Polymer composites for various biomedical applications. Ceram Int.

[22] Otgonbayar Z, Oh W. Insights into 2D MXenes for versatile Biomedical Applications. In *Bionanomaterials for biosensors, drug delivery, and medical applications*.: CRC: 177–210.

[23] Hu B, Ouyang Y, Zhao T, Wang Z, Yan Q, Qian Q, Wang W, Wang S. Antioxidant hydrogels: antioxidant mechanisms, design strategies, and applications in alleviating oxidative stress-related diseases. Adv Healthc Mater 2024:2303817. 10.1002/adhm.202303817 38166174

[24] Zhao Y, Ran B, Lee D, Liao J. Photo-Controllable Smart Hydrogels for Biomedical Application: a review. Small Methods 2023:2301095. 10.1002/smtd.202301095 37884456

[25] Jiang P, Li Q, Luo Y, Luo F, Che Q, Lu Z, Yang S, Yang Y, Chen X, Cai Y. Current status and progress in research on dressing management for diabetic foot ulcer. Front Endocrinol (Lausanne) 2023, 14. 10.3389/fendo.2023.1221705 PMC10470649 37664860

[26] Lee J, Parthiban P, Jin G, Knowles JC, Kim H (2021). Materials roles for promoting angiogenesis in tissue regeneration. Prog Mater Sci.

[27] Fuchs S, Shariati K, Ma M (2020). Specialty tough hydrogels and their biomedical applications. Adv Healthc Mater.

[28] Park H, Otte A, Park K (2022). Evolution of drug delivery systems: from 1950 to 2020 and beyond. J Control Release.

[29] Zhang X, Tan B, Wu Y, Zhang M, Xie X, Liao J (2022). An injectable, self-healing carboxymethylated chitosan hydrogel with mild photothermal stimulation for wound healing. Carbohydr Polym.

[30] Wang L, Hu P, Jiang H, Zhao J, Tang J, Jiang D, Wang J, Shi J, Jia W (2022). Mild hyperthermia-mediated osteogenesis and angiogenesis play a critical role in magnetothermal composite-induced bone regeneration. Nano Today.

[31] Thapa K, FitzSimons TM, Otakpor MU, Siller MM, Crowell AD, Zepeda JE, Torres E, Roe LN, Arts J, Rosales AM (2023). Photothermal Modulation of Dynamic Covalent Poly (ethylene glycol)/PEDOT composite hydrogels for On-Demand drug delivery. ACS Appl Mater Interfaces.

[32] El-Aswar EI, Ramadan H, Elkik H, Taha AG (2022). A comprehensive review on preparation, functionalization and recent applications of nanofiber membranes in wastewater treatment. J Environ Manage.

[33] Thakur A, Chandran BSN, Davidson K, Bedford A, Fang H, Im Y, Kanduri V, Wyatt BC, Nemani SK, Poliukhova V. Step-by‐step guide for synthesis and delamination of Ti3C2Tx MXene. Small Methods 2023:2300030. 10.1002/smtd.202300030 37150839

[34] Noshadi I, Hong S, Sullivan KE, Sani ES, Portillo-Lara R, Tamayol A, Shin SR, Gao AE, Stoppel WL, Black LD (2017). In vitro and in vivo analysis of visible light crosslinkable gelatin methacryloyl (GelMA) hydrogels. Biomater Sci.

[35] Li S, Deng Q, Zhang Y, Li X, Wen G, Cui X, Wan Y, Huang Y, Chen J, Liu Z (2020). Rational design of conjugated small molecules for superior photothermal theranostics in the NIR-II biowindow. Adv Mater.

[36] Tao B, Lin C, Yuan Z, He Y, Chen M, Li K, Hu J, Yang Y, Xia Z, Cai K (2021). Near infrared light-triggered on-demand cur release from Gel-PDA@ Cur composite hydrogel for antibacterial wound healing. Chem Eng J.

[37] Liu T, Liu S, Shi Y, Zhang Z, Ding S, Hou K, Zhang W, Meng X, Li F (2024). Electrospun nanofiber membranes for rapid liver hemostasis via N-alkylated chitosan doped chitosan/PEO. Int J Biol Macromol.

[38] Jeyagaran A, Lu C, Zbinden A, Birkenfeld AL, Brucker SY, Layland SL. Type 1 diabetes and engineering enhanced islet transplantation. Adv Drug Deliv Rev 2022:114481. 10.1016/j.addr.2022.114481 PMC9531713 36002043

[39] Vo TS, Vo TTBC, Nguyen TS, Tien TT (2021). Fabrication and characterization of Gelatin/Chitosan Hydrogel Membranes. J Turkish Chem Soc Sect A: Chem.

[40] Sethi S, Kaith BS. A review on chitosan-gelatin nanocomposites: synthesis, characterization and biomedical applications. Reactive Funct Polym 2022:105362.

[41] Liang Y, He J, Guo B (2021). Functional hydrogels as wound dressing to enhance wound healing. ACS Nano.

[42] Guo B, Dong R, Liang Y, Li M (2021). Haemostatic materials for wound healing applications. Nat Rev Chem.

[43] Lin F, Duan Q, Wu F (2020). Conjugated polymer-based photothermal therapy for killing microorganisms. ACS Appl Polym Mater.

[44] Chen L, Sun X, Cheng K, Topham PD, Xu M, Jia Y, Dong D, Wang S, Liu Y, Wang L (2022). Temperature-regulating Phase Change Fiber Scaffold toward mild photothermal–chemotherapy. Adv Fiber Mater.

[45] Yang Z, Huang R, Zheng B, Guo W, Li C, He W, Wei Y, Du Y, Wang H, Wu D (2021). Highly stretchable, adhesive, biocompatible, and antibacterial hydrogel dressings for wound healing. Adv Sci (Weinh).

[46] Li Z, Bai H, Jia S, Yuan H, Gao L, Liang H (2021). Design of functional polymer nanomaterials for antimicrobial therapy and combatting resistance. Mater Chem Front.

[47] Thambirajoo M, Maarof M, Lokanathan Y, Katas H, Ghazalli NF, Tabata Y, Fauzi MB (2021). Potential of nanoparticles integrated with antibacterial properties in preventing biofilm and antibiotic resistance. Antibiotics.

[48] Zhu W, Mei J, Zhang X, Zhou J, Xu D, Su Z, Fang S, Wang J, Zhang X, Zhu C (2022). Photothermal Nanozyme-based Microneedle Patch against Refractory bacterial biofilm infection via Iron‐Actuated Janus Ion Therapy. Adv Mater.

[49] Srinivasan R, Santhakumari S, Poonguzhali P, Geetha M, Dyavaiah M, Xiangmin L (2021). Bacterial biofilm inhibition: a focused review on recent therapeutic strategies for combating the biofilm mediated infections. Front Microbiol.

[50] Bello AB, Kim D, Kim D, Park H, Lee S (2020). Engineering and functionalization of gelatin biomaterials: from cell culture to medical applications. Tissue Eng Part B: Reviews.

[51] Yanli Z, Jiayao M, Chunqing Z, Yuting Z, Zhiyan Z, Yulin Z, Minghan L, Longquan S, Dehong Y, Wenjuan Y. MY-1 loaded Nano‐Hydroxyapatite Accelerated Bone regeneration by increasing type III Collagen Deposition in early‐stage ECM via a Hsp47 dependent mechanism. Adv Healthc Mater 2023:2300332. 10.1002/adhm.202300332 36999955

[52] Jiang X, Zhou T, Wang Z, Qi B, Xia H (2017). HSP47 promotes glioblastoma stemlike cell survival by modulating tumor microenvironment extracellular matrix through TGF-β pathway. ACS Chem Neurosci.

[53] Krüger-Genge A, Blocki A, Franke R, Jung F (2019). Vascular endothelial cell biology: an update. Int J Mol Sci.

[54] Tong L, Liao Q, Zhao Y, Huang H, Gao A, Zhang W, Gao X, Wei W, Guan M, Chu PK (2019). Near-infrared light control of bone regeneration with biodegradable photothermal osteoimplant. Biomaterials.

[55] Liang Z, Luo J, Liu S, Gu Y, Cui Z, Zhu Y, Yu Z, Zhao X, Guo B, Song B (2023). Injectable, antibacterial, ROS scavenging and pro-angiogenic hydrogel adhesives promote chronic wound healing in diabetes via synergistic release of NMN and Mg2+. Chem Eng J.

[56] Guan Y, Niu H, Liu Z, Dang Y, Shen J, Zayed M, Ma L, Guan J (2021). Sustained oxygenation accelerates diabetic wound healing by promoting epithelialization and angiogenesis and decreasing inflammation. Sci Adv.

[57] Peng Y, Wu S, Li Y, Crane JL (2020). Type H blood vessels in bone modeling and remodeling. Theranostics.

[58] He Y, Sun MM, Zhang GG, Yang J, Chen KS, Xu WW, Li B (2021). Targeting PI3K/Akt signal transduction for cancer therapy. Signal Transduct Target Ther.

[59] Harsha L, Brundha MP. Role of collagen in wound healing. Drug Invention Today 2020, 13.

[60] Wang H, Xu Z, Zhao M, Liu G, Wu J (2021). Advances of hydrogel dressings in diabetic wounds. Biomater Sci.

