# Supplementary material for: NIR-responsive electrospun nanofiber dressing promotes diabetic-infected wound healing with programmed combined temperature-coordinated photothermal therapy
Source: J Nanobiotechnology. 2024 Jul 1;22:384. doi: 10.1186/s12951-024-02621-2 (PMC11218286; doi:10.1186/s12951-024-02621-2)
Supplement: Supplementary file 1 — Supplementary Material 1 [file 12951_2024_2621_MOESM1_ESM.docx]

Supplementary material


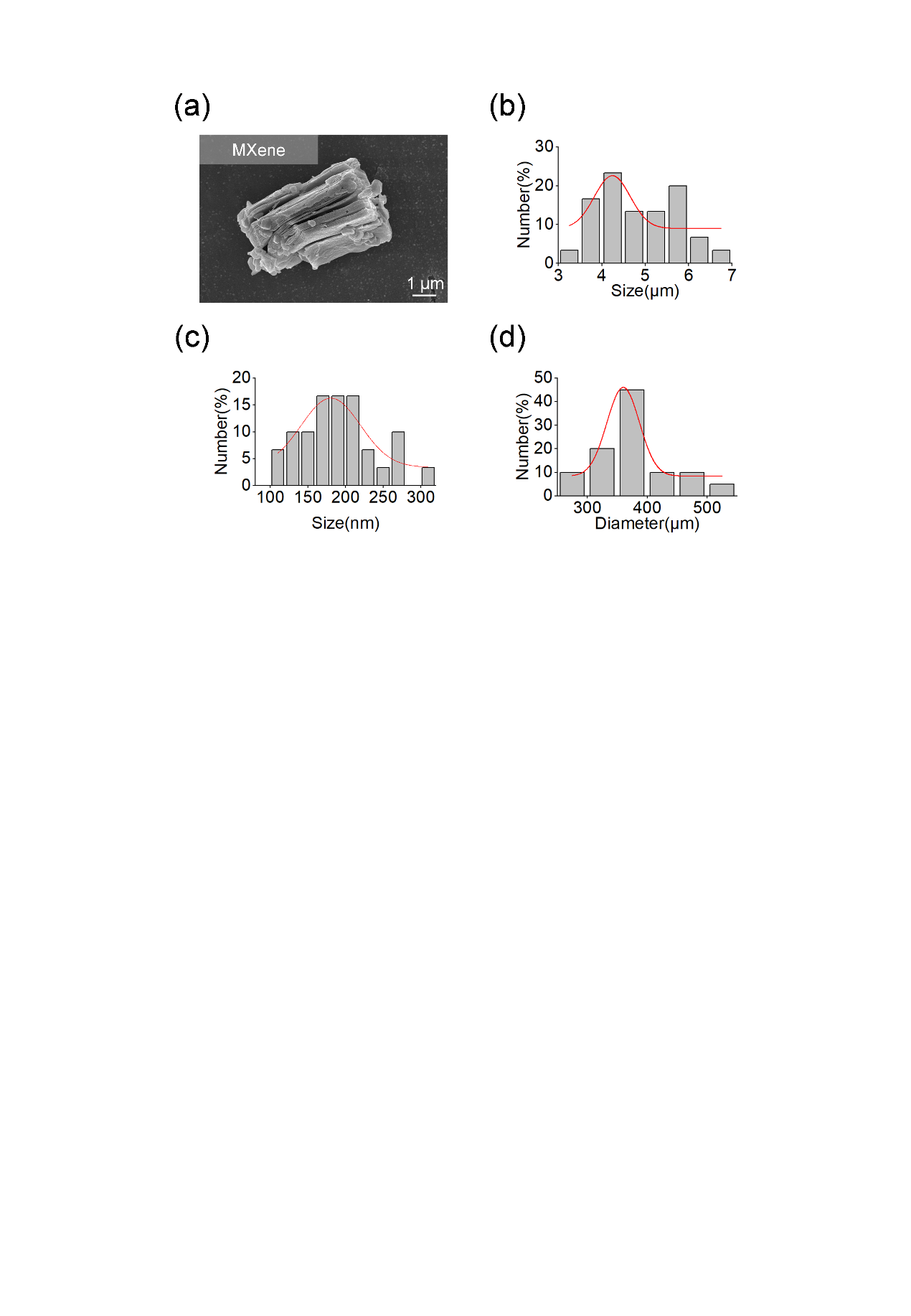


**Fig. S1** Characterization of MXene and GelMA@ MXene: (a) Scanning electron microscopy (SEM) images of MXene. (b) Size distribution of the initially synthesized MXene. (c) Size distribution of MXene following a 12-hour ultrasonic treatment. (d) Size distribution of GelMA@ MXene photothermal microspheres.

**
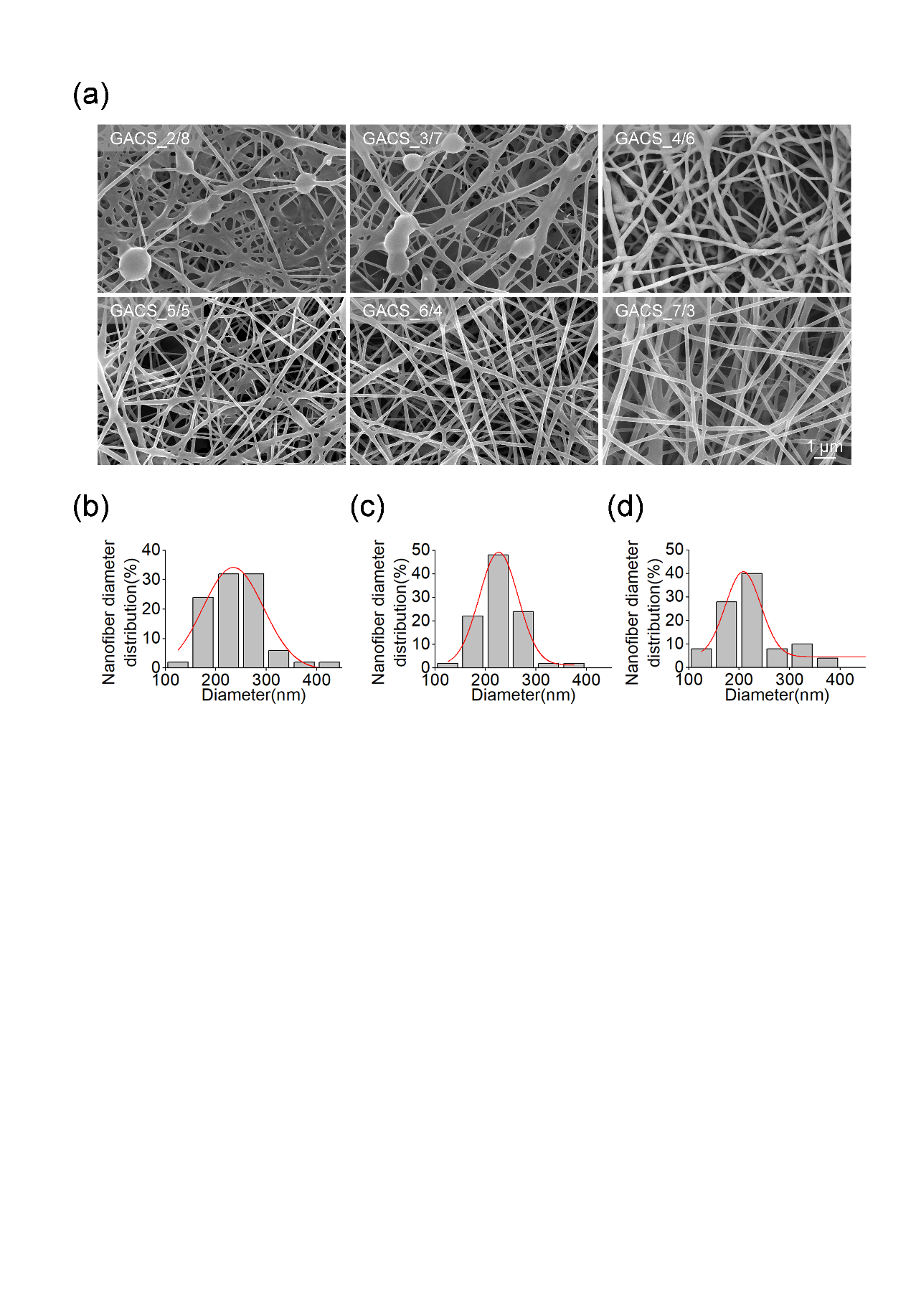
**

**Fig. S2** Characterization of gelatin/chitosan nanofiber membranes: (a) Scanning electron microscopy (SEM) images of gelatin/chitosan nanofiber membranes with different weight ratios. (b-d) CS, GACS and MGACS nanofibrous membranes diameter distribution.


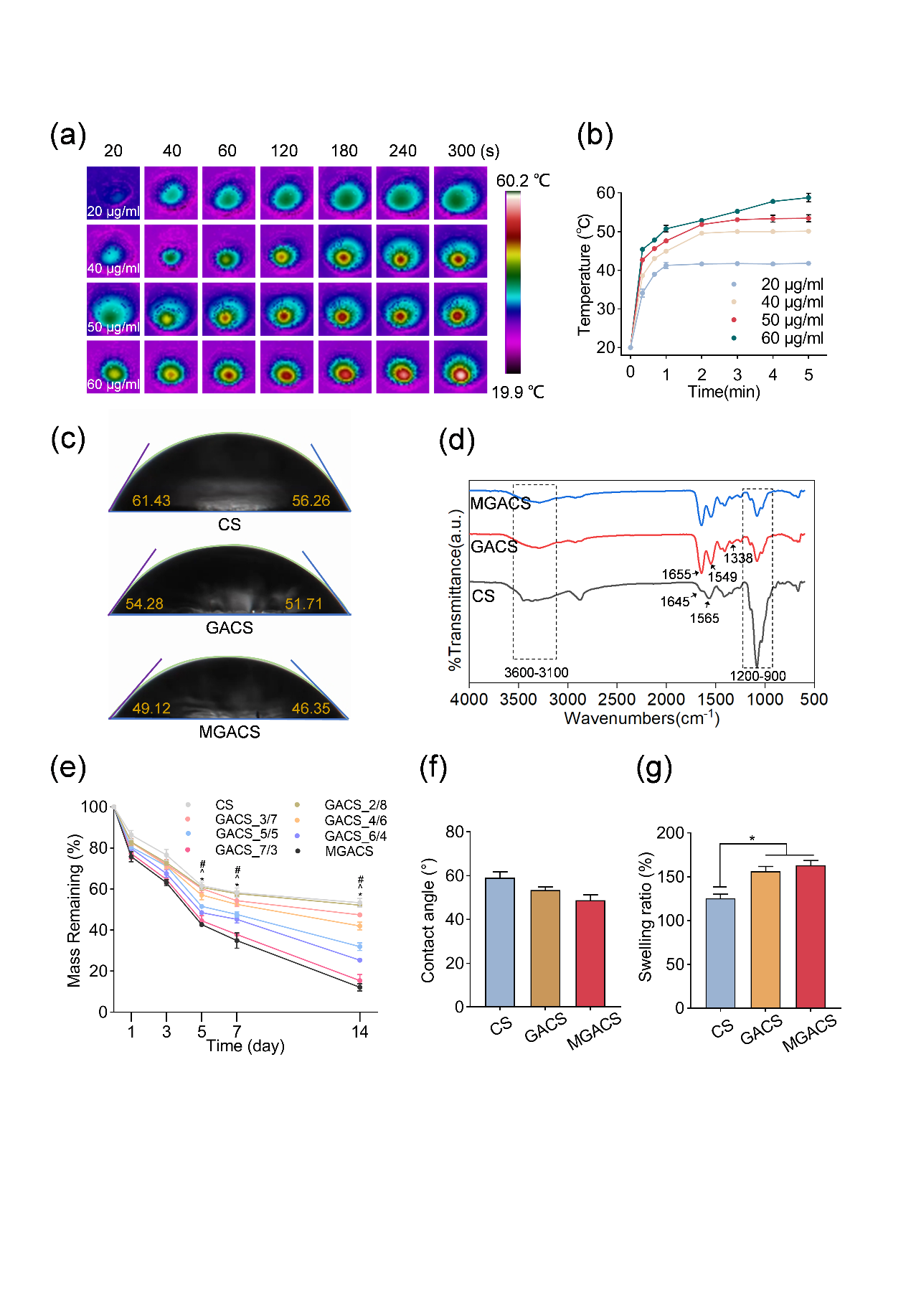


**Fig. S3** (a) Near-Infrared (NIR) imaging of GelMA@MXene photothermal microspheres at concentrations of 20, 40, 50, and 60 μg/ml under irradiation of 808 nm at 1.0 W/cm^2^. (b) Temperature change curves of GelMA@MXene photothermal microspheres at various concentrations under 1.0 W/cm^2^ near-infrared light (NIR) irradiation. (c,f) Contact angles of CS, GACS, and MGACS nanofiber membranes. (d) FT-IR spectra of CS, GACS, and MGACS nanofiber membranes. (e) Degradation profiles of nanofiber membranes. (g) Degree of swelling in nanofiber membranes at different weight ratios (%).


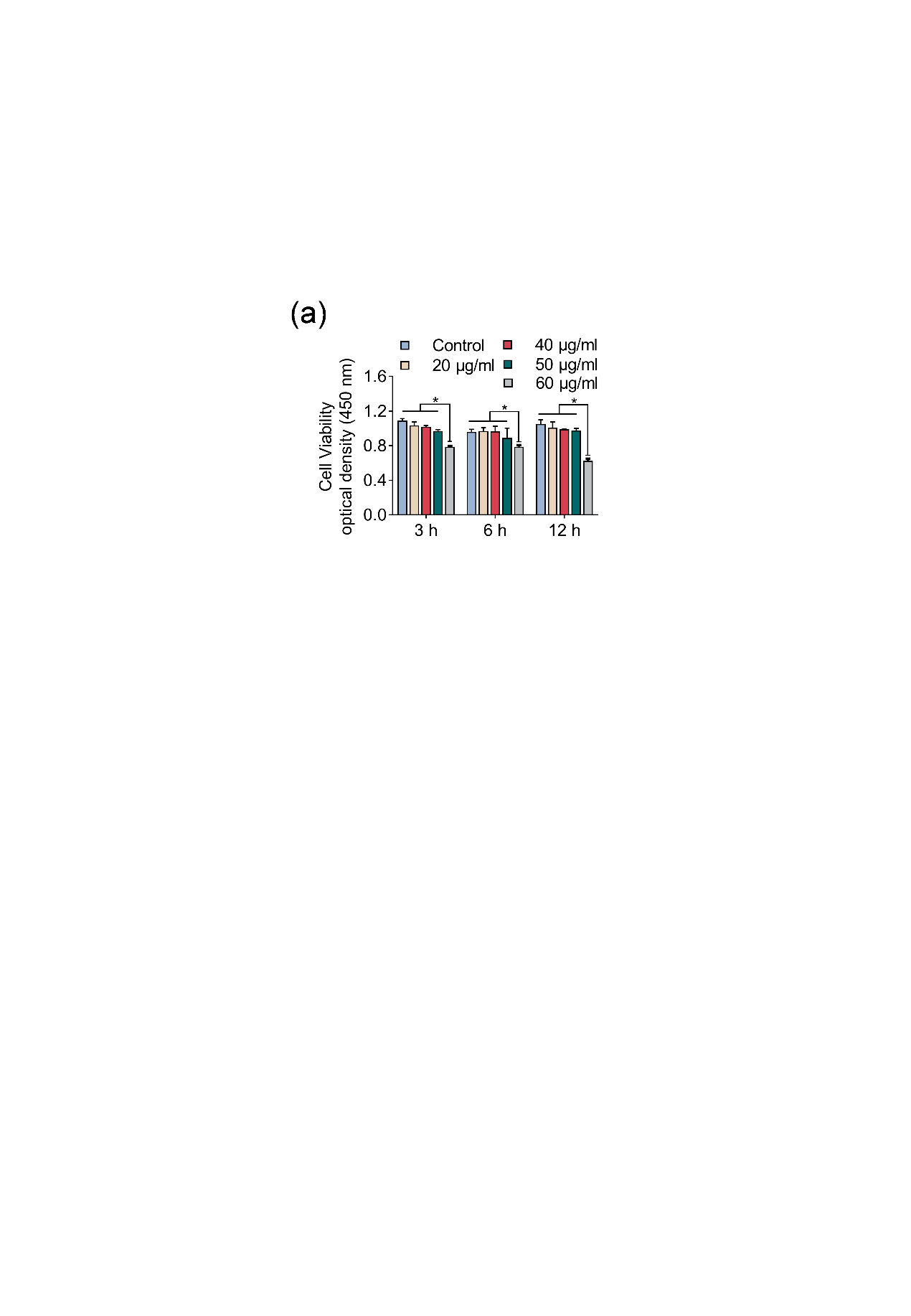


**Fig. S4** (a) L929 cells, post-treatment with photothermal microspheres at concentrations of 20, 40, 50, and 60 μg/ml, were subjected to CCK-8 assays. (n = 3).


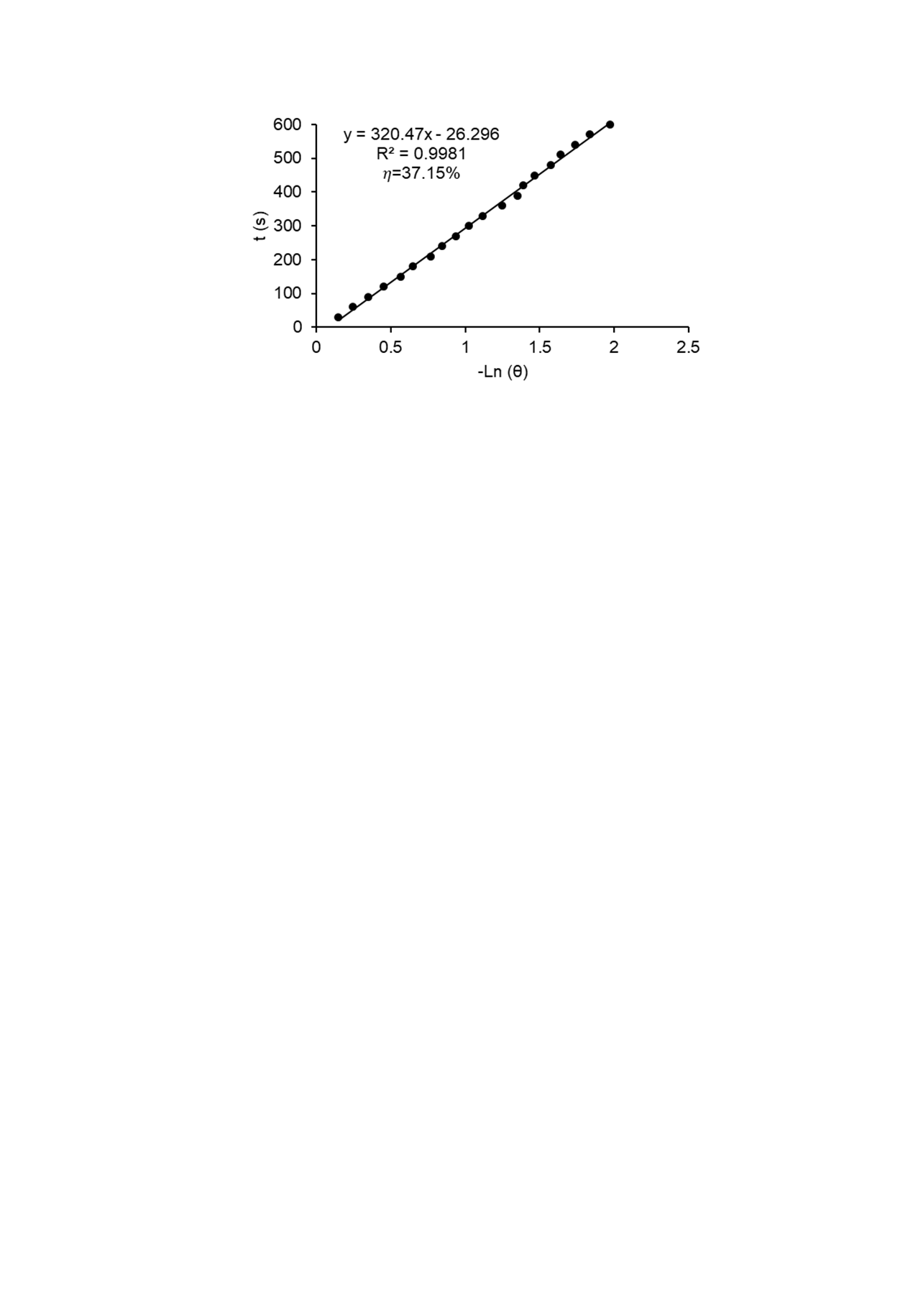


**Fig. S5** Photothermal performance of MGACA nanofiber membranes by cooling to room temperature with linear analysis.
